# An Elucidative Review of the Nanomaterial Effect on the Durability and Calcium-Silicate-Hydrate (C-S-H) Gel Development of Concrete

**DOI:** 10.3390/gels9080613

**Published:** 2023-07-28

**Authors:** Farqad Yousuf Al-saffar, Leong Sing Wong, Suvash Chandra Paul

**Affiliations:** 1 Department of Civil Engineering, College of Engineering, Universiti Tenaga Nasional, Jalan IKRAM-UNITEN, Kajang 43000, Selangor, Malaysia; 2 Institute of Energy Infrastructure, Universiti Tenaga Nasional, Jalan IKRAM-UNITEN, Kajang 43000, Selangor, Malaysia; 3 Department of Civil Engineering, International University of Business Agriculture and Technology, 4 Embankment Drive Road, Sector 10, Dhaka 1230, Bangladesh

**Keywords:** nanomaterial, concrete, durability, energy savings, C-S-H gel

## Abstract

Concrete as a building material is susceptible to degradation by environmental threats such as thermal diffusion, acid and sulphate infiltration, and chloride penetration. Hence, the inclusion of nanomaterials in concrete has a positive effect in terms of promoting its mechanical strength and durability performance, as well as resulting in energy savings due to reduced cement consumption in concrete production. This review article discussed the novel advances in research regarding C-S-H gel promotion and concrete durability improvement using nanomaterials. Basically, this review deals with topics relevant to the influence of nanomaterials on concrete’s resistance to heat, acid, sulphate, chlorides, and wear deterioration, as well as the impact on concrete microstructure and chemical bonding. The significance of this review is a critical discussion on the cementation mechanism of nanoparticles in enhancing durability properties owing to their nanofiller effect, pozzolanic reactivity, and nucleation effect. The utilization of nanoparticles enhanced the hydrolysis of cement, leading to a rise in the production of C-S-H gel. Consequently, this improvement in concrete microstructure led to a reduction in the number of capillary pores and pore connectivity, thereby improving the concrete’s water resistance. Microstructural and chemical evidence obtained using SEM and XRD indicated that nanomaterials facilitated the formation of cement gel either by reacting pozzolanically with portlandite to generate more C-S-H gel or by functioning as nucleation sites. Due to an increased rate of C-S-H gel formation, concrete enhanced with nanoparticles exhibited greater durability against heat damage, external attack by acids and sulphates, chloride diffusion, and surface abrasion. The durability improvement following nanomaterial incorporation into concrete can be summarised as enhanced residual mechanical strength, reduced concrete mass loss, reduced diffusion coefficients for thermal and chloride, improved performance against sulphates and acid attack, and increased surface resistance to abrasion.

## 1. Introduction

Improving concrete durability was and is still a subject of interest for scientists and engineers [1,2]. Many approaches and methods were used to increase concrete’s resistance against threats from natural weathering, chemical attacks, surface abrasion, and fire exposure. Concrete durability problems compromise its structural integrity, leading to economic and human losses [3]. Several measures were taken to alleviate the problems [4,5,6], including but not limited to the following: a decrease in the water-to-cement ratio (w/c), incorporation of supplementary cementitious materials (SCMs) to partially replace cement, application of polymers, and utilization of surface coating substances. By reducing porosity, creating discontinuous pore networks, and strengthening the interfacial transition zone (ITZ) in concrete [7,8], there is a significant enhancement in the durability of concrete. Furthermore, enhancing the mechanical characteristics of concrete and compacting its microstructure can significantly decrease its permeability and ability to absorb water [9]. Pore interconnectivity directly determines the permeability of concrete; the more interconnected the pore system, the greater the permeability and water diffusion. Pore connectivity likely decreases with increases in the degree of cement hydration, owing to a growing solid phase in the cement matrix, which is represented by hydration products (e.g., calcium silicate hydrate (C-S-H), portlandite (CH), ettringite (AFt), and monosulfoalmonate (AFm)). According to Hewlett and Liska [10], the gel that contributes to the strength characteristics of concrete is a porous and amorphous compound called calcium silicate hydrate (C-S-H). The nanostructured, gel-like hydrate bridges the unreacted cement particles as a connected network of solids into the empty spaces (pores) and is thereby considered the principal binding phase among other hydrates. The development of C-S-H gel greatly reduces the permeability of concrete and, as a result, enhances its endurance.

Incorporating ultra-fine materials (e.g., 1–100 nm) such as nanoparticles is among the most influential methods to increase the mechanical strength of concrete and perfect its microstructure [11,12,13,14]. Recent studies and published papers demonstrated that nanomaterials play an advanced role in improving concrete’s compressive strength, tensile strength, and flexural strength [15,16]. For example, nanosilica [17], which is regarded as the earliest applied and most common nanomaterial in concrete, had a remarkable effect by enhancing the performance of the material with high mechanical and durability properties. This can be attributed to its high surface area, which provides seeds for hydration products, and to its nano-filling effect on reducing the pores in concrete. Thereby, nanosilica can dramatically reduce the permeability of concrete. Nanosilica materials enhance the microstructure not only because of the nanofiller effect but also through pozzolanic reactivity, which in turn promotes cement hydration, formatting a higher amount of C-S-H gel [18]. Other nanomaterials such as nanoclay, titanium oxide, nanometakaolin, nanoC-S-H, nanoCaCO_3_, carbon nanotube, etc., improve concrete microstructure with filling and seeding effects [11,12,13,14]. According to the literature, nanoparticles enhance the formation of C-S-H gel with a compact structure by filling its nanopores, as discussed in Section 2. Some nanoparticles can provide nucleation sites owing to their super fineness, thus accelerating the precipitation and formation of C-S-H gel. Owing to the high efficiency of nanomaterials in improving the mechanical properties of cementitious materials, the applications of nanoparticles were broadened to include ultra-high performance concrete [13], self-compacted concrete [19], reactive powder concrete [20], heavyweight concrete [21], marine concrete [22], shotcrete [23], green concrete [24], recycled aggregate concrete [25], lightweight concrete [26], high-volume fly ash concrete [27], carbon fibre-reinforced concrete [28], and foam concrete [29].

The impact of numerous nanomaterials, such as nanosilica, nanoZnO, nanoFe_2_O_3_, nanoAl_2_O_3_, and nanoTiO_2_, on the properties of concrete was discussed in several published works [11,12,13,14]. The results confirmed the ability of these ultra-fine materials to ameliorate the cementitious materials’ macroscopic characteristics and functioning. However, due to the agglomeration effect of nanomaterials, these nanoparticles need to be optimised to reduce their negative impact and efficiency in cementitious materials. Hence, several studies were conducted to determine the optimum dosage for better performance in cementitious materials. Nanotechnology in concrete has always been an area of focus for concrete technologists, and due to recent advances in nanomaterials, researchers have extended their investigations to include hybrid nanoparticles [30], nanoparticles with fibres [28], and nanoparticles with cementitious supplementary materials [21]. The incorporation of nanomaterials in concrete helped enhance its permeability properties by mitigating the material’s water absorption and hindering the penetration and transport of deleterious ions [30]. This is attributed to nanoparticles’ role in reducing pore size and connectivity by advancing the growth of C-S-H gel. Various studies investigated the influence of incorporating nanomaterials on the durability performance of concrete [11,12,13,14]. Hence, in this article, a comprehensive review was completed to highlight the effect of nanomaterials on cement gel in concrete and how their durability performance can be enhanced against heat, acid attack, sulphate attack, chloride attack, and surface abrasion.

This review article demonstrates the mechanism of action underlying how nanoparticles improve the durability properties of concrete by improving the hydration of binding gel and other hydration products. The improvement in concrete durability following nanomaterial incorporation can be summarised as enhanced residual mechanical strength, reduced concrete mass loss, reduced diffusion coefficients for thermal and chloride, improved performance against sulphates and acid attack, and increased surface resistance to abrasion. The technological progress in nanoconcrete is in line with the ninth United Nations Sustainable Development Goal, which is ‘Industry, Innovation, and Infrastructure’. This review was performed by referring to the most up-to-date studies and highlighting their results to draw a robust conclusion. It provides useful ideas for future published research works related to the role of nanomaterials in improving concrete durability. Despite the literature having considerable review articles related to the effects of nanoparticles on the durability of cement-based composites, there is a lack of published reviews explaining the efficiency of nanomaterials in promoting C-S-H gel formation, which has a considerable impact on concrete’s resistance to heat, acid, sulphate, chloride, and surface abrasion.

## 2. Microstructure of Concrete and C-S-H Gel Hydration with Nanomaterials

### 2.1. Development of C-S-H Gel in Concrete Induced with Nanomaterials

Several studies have shown that the addition of nanoparticles can greatly enhance the strength and durability of concrete by improving the hydration of tricalcium silicate (C_3_S) found in cement. This is achieved as the C-S-H gel expands into the capillary pores, binding the solid products and sealing any microcracks that may impact the structural integrity of the concrete [13,30,31,32]. The exact mechanisms behind the improvement in hydration using nanomaterials have been studied extensively, and multiple theories have been proposed to explain their effectiveness [33]. An example of a material with a significant pozzolanic impact is silica nanoparticles. They interact with calcium hydroxide (CH), which encourages the expansion of the C-S-H gel, resulting in an improvement. Additionally, because of its sizeable specific area, nanosilica can act as a reactive siliceous surface, aiding in early C-S-H growth. Moreover, silica nanoparticles can serve as seeds that facilitate cement hydration and densify the gel structure by blocking the voids between C-S-H particles and thereby increasing its stiffness.

Incorporating C-S-H seeds into cementitious materials has proved to be effective in improving the early strength properties of concrete by accelerating the precipitation of silicates [34]. It was also discovered that the Ca/Si ratio in the C-S-H seeds had a significant impact on the effectiveness of the cement acceleration process, and a low Ca/Si ratio in C-S-H seeds further reduced the setting time of cement, shortening the induction period of the cement hydration process [35]. This could be attributed to the large surface areas provided by the low Ca/Si gel, which facilitated hydration. Land et al. [35] pointed out that the chemical composition and particle size of C-S-H seeds were the two primary factors that determined the effectiveness of the water–cement reaction. Moreover, incorporating nano-C-S-H particles with mineral admixtures hastened their secondary hydration process [36].

The introduction of nanoclay encouraged the production of C-S-H and carboaluminate (C-A-S-H) gels following its reaction with various cement components such as C_3_S, C_3_A, and C_4_AF. In addition, a rise in the pore solution’s Ca^2+^ ionic concentration was detected, and less consumption of silicate phases occurred in the hydration process. Microstructural observation of concrete [37] revealed that calcined nanoclay can refine the microstructure of concrete by filling in pores and exhibiting pozzolanic behaviour. This reaction occurs with Portlandite and results in the production of more C-S-H gel. Graphene oxide sheets promoted the formation of ettringite as flower-like crystals, while there was a dramatic consumption of silicates and an increase in Ca^2+^ ions with nanosilica incorporation [31]. Nanotitanium dioxide had a lower cement hydration acceleration compared to other nanomaterials and played an inert role in hydration to some extent [31]. The assumption was emphasised by the lower consumption of silicate phases. On the other hand, nanotitanium dioxide promoted the microstructure by stimulating more C-S-H gel and preventing CH growth, in addition to its filling and nucleation effects [38].

Nanometakaolin can generate additional quantities of C-S-H gel due to nanoparticles’ role in catalysing the cement–water reaction. It was reported that nanometakaolin has the ability to increase the chemical bonding and cohesion forces in the C-S-H structure. Like nanosilica, nanometakaolin is a pozzolanic material that creates more C-S-H gel through a secondary reaction with CH crystals [39]. The inclusion of nano-ZrO_2_ particles [40] can also improve the cement reaction with their nucleation effect in a similar manner to nanosilica. Devi et al. [41] discovered that C-S-H gel with graphene oxide was less porous because of its denser microstructure. This can be attributed to embedded CH crystals and ettringite needles while having interlocked and interweaved foil features. Observing the formation of massive and denser hydrated crystals revealed that graphene oxide can function as a concrete reinforcement agent. XRD results indicated that blending graphene oxide with cement assisted the hydration of unreacted cement particles at later ages, making the concrete stronger and more durable. Moreover, carbon nanotubes [42] can also facilitate C-S-H hydration by providing nucleation sites because of their higher surface area. Microstructure observation confirmed that the functional group (carboxyl) of carbon nanotubes can interact with CH, generating an additional amount of gel hydrates. On the other hand, it was reported that higher amounts of carbon nanotubes limited the hydration of C-S-H gel because they reduced the amount of free water, which is crucial for hydration to proceed. Furthermore, carbon nanotubes cover the unreacted cement particles, hindering water diffusion and preventing further reaction. Hence, the incorporation of carbon nanotubes can have a negative impact on cement hydration. Meanwhile, the size and shape of carbon nanotubes, in addition to their surface functional groups, are the major factors influencing their hydration effect. Wang et al. [43] used Si NMR spectroscopy to examine how nanofillers changed the structure of C-S-H gels.

The addition of nanofillers such as nanosilica has two major effects on the hydration of C-S-H gel. Firstly, they provide nucleation sites and undergo a pozzolanic reaction with CH hydrates. Secondly, their high water absorption capacity reduces the amount of proton water in the C-S-H gel, shortening the distance between the Ca, O, and Si atoms. Additionally, the nanofillers also break the Si-O-Si spatial structure of silicate tetrahedrons in the C-S-H gel and cause hydroxylation of the tetrahedron. These newly produced Si-OH monomers interact with one another and condense to create a higher polymerization silicate tetrahedron. Furthermore, one study found that incorporating fillers into a C-S-H gel reduced the amount of proton water in the gel, which, in turn, increased the strength of the chemical bond (ionic bond and covalent binding) between the gel’s Ca, O, and Si atom structural groups. However, the nanofillers’ excessive absorption of gel water could inhibit further hydration, and this could be indicated as an unfavourable phenomenon. Several comparison studies [12] confirmed that nanomaterials had a more effective role in improving cement hydration than supplementary cementitious materials (SCMs) when the pozzolanic reactivity of nanomaterials such as nanosilica outperformed common SCMs. Typically, the majority of nanoparticles aid in the process of cement gel hydration by creating more locations for nucleation and supporting the development of C-S-H gel through nucleation and growth processes. This was confirmed by Long et al. [44], who measured the heat released to different nanomaterial binders and found that reducing the period of the induction stage and the time for the main peak in heat flow by nanosilica and nano-C-S-H were indications of the nanoparticle effect on the rate of cement hydration. Higher heat release rate peak values were recorded with these types of nanoparticle binders. The study concluded that nanosilica and nano-C-S-H can remarkably increase the rate of hydrate nucleation and growth, while the effect was greater with increasing the rate of nucleation. Nano-CaCO_3_ [45] had no effective role either in the nucleation rate or growth rate of hydration, and that was attributed to its low chemical activity. The effect of nanoparticles on cement hydration was dominated by the particle’s size, affinity, and chemical activity. The C-S-H gels appear in a great number of clusters with a more uniform distribution in the presence of nanoparticles, binding various substances such as CH and unreacted cement particles with aggregates in concrete. This characteristic of C-S-H gel helps fill more voids at the microscopic level, making the microstructure of concrete more compact and homogeneous. By enhancing the concrete microstructure, durability will be significantly improved.

### 2.2. Microstructure of Concrete with Nanomaterials

Numerous microstructure observation techniques (e.g., SEM, XRD, and TGA) were used to investigate the kinetics of nanomaterials in cementitious materials and their mechanism in densifying and compacting the concrete microstructure [46,47]. The microstructure of concrete is primarily improved with nanoparticles through three effects, namely the nanofiller effect, pozzolanic reactivity, and the nucleation effect [48]. The nanofiller effect is attributed to nanoparticles’ ultra-small size and higher surface area, which helps them to occupy a higher percentage of fine voids in concrete. Nanoparticles work as crystal nuclei, promoting cement hydration. In addition, nanoparticles generate more C-S-H gel through their reaction with CH. These effects remarkably enhance the microstructural packing efficiency of concrete and subsequently improve the material’s durability and mechanical strength.

**Figure 1:**
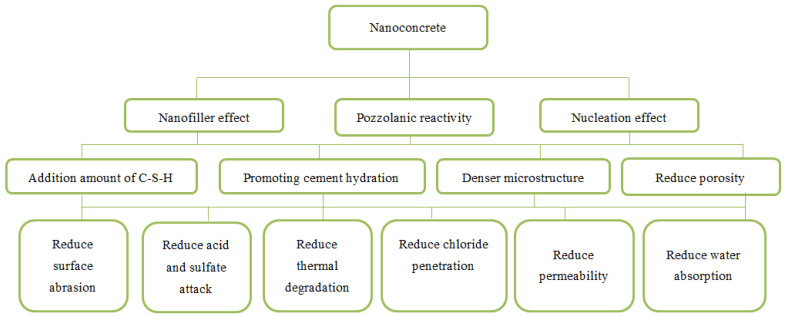
The action by which nanoparticles enhance the durability of concrete.

Figure 1 shows a diagram that summarises the mechanism by which nanoparticles improve the durability of concrete. Several scanning electron microscopic (SEM) images from different studies presented a denser, homogeneous, and more uniform microstructure of nano-modified concrete [11,12,13,14,15,16,17,18,19,20,21,22,23,24,25,26,27,28,29,30,31,32,33,34,35,36,37,38,39,40,41,42,43,44,45,46,47,48]. The interfacial transition zone (ITZ) was also improved with the addition of nanoparticles owing to their microaggregate filling effect [49,50]. This microstructure improvement implies that there was a significant increase in the concrete’s resistance to acid and sulphate attack and a reduction in its permeability and water absorption. The SEM images demonstrated that the concrete microstructure containing nanoparticles exhibited denser textures and showed fewer destructive effects and microcracks due to the nanoparticle mechanism of action for advancing the growth of C-S-H gel [30,31,32,33,34,35,36,37,38,39,40,41,42,43,44,45]. Fewer CH crystals were observed in all types of nanomaterials incorporating concrete with higher amounts of gel-like hydrates. Even at higher temperatures, the microstructure of nanoconcrete was denser at 200 °C, more homogeneous at 400 °C, and had fewer cavities at 700 °C. Moreover, the concrete microstructure exhibited fewer microcracks at 400 and 600 °C.

**Figure 2:**
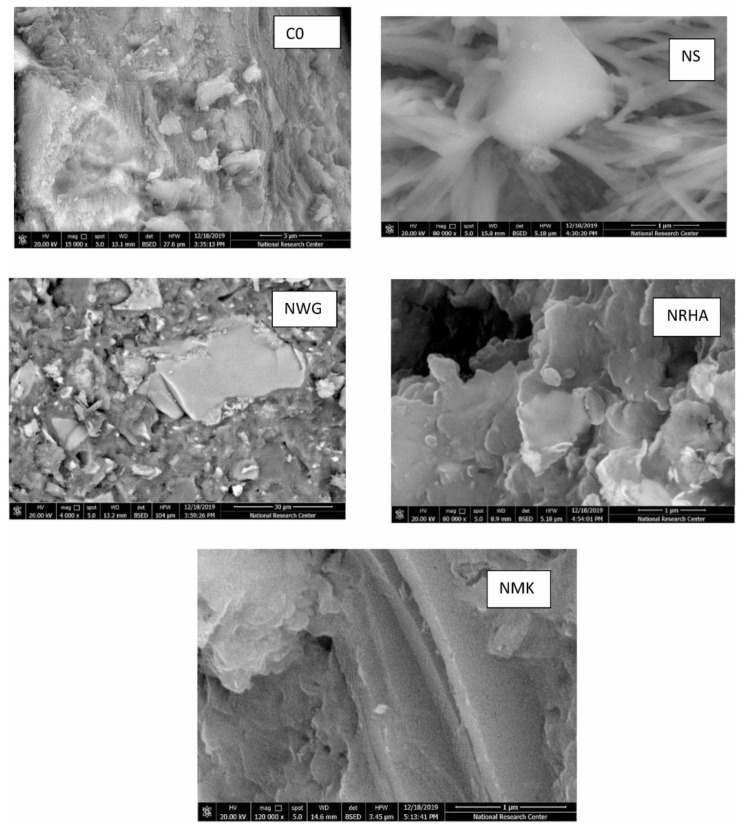
SEM images showing concrete with nanoparticles. Reproduced with permission from [51] (*Journal of Building Engineering*); published by (Elsevier), (2021). (Note: milled nanometakaolin (NMK), nanowaste glass (NWG), nano rice husk ash (NRHA), nanosilica (NS), control concrete (C0)).

Figure 2 shows SEM images of concrete with nanoparticles [51]. The micrographs from several SEM studies indicated that the hydration products in the nanoconcrete microstructure were “distributed neatly and tidy, and the texture was denser with no obvious aciculate AFt or cubic CH to be traced” [52]. Furthermore, using the SEM images, it was observed that the microstructure of nanomodified concrete exhibited a lower amount of CH, gypsum, and ettringite needle crystals, in addition to a denser and more homogenous structure. X-ray diffraction (XRD) tests on nanoconcrete were performed and published in several research articles [11,12,13,14,15,16,17,18,19,20,21,22,23,24,25,26,27,28,29,30,31,32,33,34,35,36,37,38,39,40,41,42,43,44,45,46,47,48]. All the test results indicated a higher intensity of the C-S-H gel peak and a lower intensity of CH for concrete in the presence of nanomaterials.

**Figure 3:**
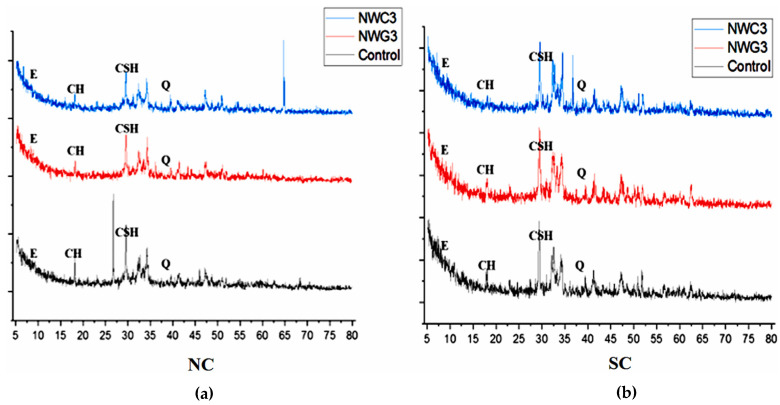
XRD patterns for 28-day self-compacted concrete under (**a**) normal curing (NC) and (**b**) self-curing (SC). Q = unhydrated SiO_2_, E = ettringite, CSH = calcium silicate hydrate, and CH = calcium hydroxide. Reproduced with permission from [53] (*Journal of Building Engineering*); published by (Elsevier), (2022).

At a temperature of 200 °C, it was observed that nanoconcrete contained smaller CH crystals. The presence of such crystals indicated the pozzolanic reactivity of nanomaterials, while their smaller size and orientation were consistent with past research findings. Incorporating nanoparticles into concrete led to an upsurge in the volume of C-S-H gel, ascribed to their nucleation effect that expedited cement hydration. The high surface area of nanoparticles acted as nuclei for cement hydrates, hence enabling this effect. Additionally, the reaction between nanoparticles and CH crystals brought about significant enhancement in the interfacial transition zone (ITZ) between the hardened cement paste and aggregates. The substitution of CH crystals with C-S-H gel resulted in superior performance. When testing the concrete using XRD, good pozzolanic reactivity was demonstrated through the lower intensity peaks in CH, C_3_S, and C_2_S. However, there was an increasing trend in CH crystals as the inclusion of nanomaterials increased due to poor distribution. The EDX analysis revealed that the Ca/Si molar ratio increased with the incorporation of nanomaterials, indicating the positive impact of these materials on the microstructure of cementitious materials. The usage of DSC and TGA [51] in thermal analyses showed that concrete with varying nanoparticles had increased C-S-H gel and a significant decrease in CH contents. The XRD patterns for 28-day concrete with 3 wt.% nanowaste glass (NWG) and 3 wt.% nanowaste ceramic (NWC) [53] are illustrated in Figure 3.

## 3. Improvement in the Heat Resistance of Concrete with Nanomaterials

Normal concrete as a building material has appreciable heat resistance with a very low value of thermal conductivity [54]. However, at elevated temperatures greater than 300 °C, the strength properties of concrete deteriorate, and at 600 °C, concrete loses its structural performance [55,56,57]. The deterioration mechanism for concrete exposed to elevated temperature results from a volume change with aggregate expansion (due to heat) versus cement paste shrinkage (due to water evaporation) [58]. The high temperature can cause damage to the concrete as a result of the drying out and breaking down of cement compounds. The C-S-H gel dehydrates and decomposes when concrete is exposed to elevated temperatures, while free CH loses its bound water, decomposing into calcium oxide. CH is rehydrated and expanded when exposed to moisture, generating built-in stresses and cracking [59,60]. The concrete mass loss is caused by heat due to capillary water evaporation in addition to hydrates and aggregate decomposition. When concrete is exposed to high temperatures, its mechanical features such as compressive, tensile, and flexural strengths are significantly decreased. Concrete spalling [61,62] is another great threat that can be induced by heat. The failure in sections closer to the concrete heated surface is a result of spalling caused by the interplay between the internal pore pressure and thermal stresses, which are characterised by the thermal gradient and thermal incompatibility [63]. For the purpose of improving the fire resistance of concrete, polypropylene fibres [64], light-weight aggregates [65], and supplementary cementitious materials (SCMs) [66] are used. Furthermore, nanomaterials are used in concrete to enhance the heat resistance of the material and reduce thermal degradation during exposure to elevated temperatures [67]. Table 1 summarises several studies conducted with regard to the improvement in concrete’s resistance to heat with the addition of nanomaterials. From Table 1, it can be seen that the incorporation of different types of nanomaterials can help mitigate the thermal conductivity of concrete, decrease the mass loss caused by thermal degradation, and improve the mechanical properties of concrete.

### 3.1. Improving Concrete Thermal Conductivity Using Nanomaterials

Several studies confirmed the ability of nanomaterials to improve concrete thermal conductivity. Reddy et al. [79] observed that adding ground-granulated blast furnace slag (GGBS) to concrete modified with 3 wt.% nanosilica resulted in lower thermal conductivity and greater thermal resistance properties. The steady-state box and transition method were used to assess concrete’s thermal properties. The results of the steady-state box method demonstrated that blending 3 wt.% nanosilica with GGBS concrete reduced thermal conductivity and thermal diffusivity by 40% and 26%, respectively, as compared with a conventional concrete mix. However, incorporating a higher amount of nanosilica exhibited lower efficiency. The results of the transient method indicated that the average thermal conductivity values for concrete samples cured after 28 days were reduced by 12% with 3 wt.% nanosilica incorporated into GGBS concrete. Thus, 3 wt.% was the optimum dosage of nanosilica to improve the thermal resistance of concrete. Kumar et al. [70] observed that the incorporation of 3 wt.% nanosilica into high-strength concrete retarded heat transfer and reduced the rate of thermal degradation. The thermal conductivity for all concrete mixes exhibited a decreasing trend when subjected to 200, 400, 600, and 800 °C temperatures. However, the high-strength nanosilica concrete had lower thermal conductivity compared to the control high-strength concrete. Nanosilica incorporation helped reduce the rate of heat transfer in high-strength concrete by 11%, 18%, 22%, and 15% at 200, 400, 600, and 800 °C, respectively. High-temperature conditions and their influence on the thermal conductivity coefficient of concrete were investigated by Wang [73]. The concrete thermal conductivity coefficient was reduced with increasing temperatures. C-S-H gel decomposition, in addition to capillary water evaporation caused by heat, was the reason for the decrease. Furthermore, the inclusion of nanoclay in concrete at a weight percentage of 0.3 and 0.5 resulted in an improvement in the thermal conductivity coefficient when compared to control concrete. Conversely, the thermal conductivity coefficient decreased with the addition of 0.1 wt.% nanoclay. The study revealed that the higher amount of nanoclay increased the concrete’s thermal conductivity coefficient when subjected to heat, and the cause was related to the concrete’s proportion.

### 3.2. Residual Mechanical Strength Improvements with Nanomaterials

**Figure 4:**
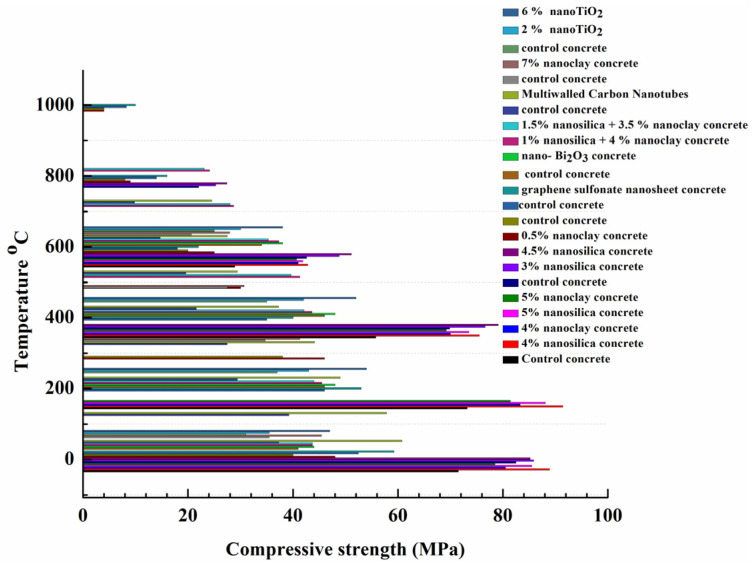
Compressive strength of concrete under different temperatures [71,73,74,75,76,77,80,81,82].

Figure 4 illustrates the collected values in the literature about concrete compressive strength under heat from [71,73,74,75,76,77,80,81,82]. The concrete compressive strength for all types, regardless of its mix proportion, exhibited a decreasing trend with increasing temperature. However, some concrete exhibited a higher compressive strength at 200 °C than at room temperature. This can be related to the influence of heat on the density of C-S-H gel in concrete, as it was reported that denser C-S-H with an increased chain length can be formed at 200 °C. The improvement in the compressive strength of concrete at high temperatures can be attributed to an increase in the van der Waals force during capillary water evaporation, thus increasing the bonding strength [70,75]. At temperatures greater than 200 °C, the compressive strength of concrete remarkably declined due to thermal degradation, and the values kept declining with increasing temperatures, reaching their lowest at 1000 °C. Furthermore, the figure emphasises the impact of nanomaterials on enhancing the residual mechanical strength of concrete, as higher values of compressive strength can be observed with nanomodified concrete than with control concrete.

**Figure 5:**
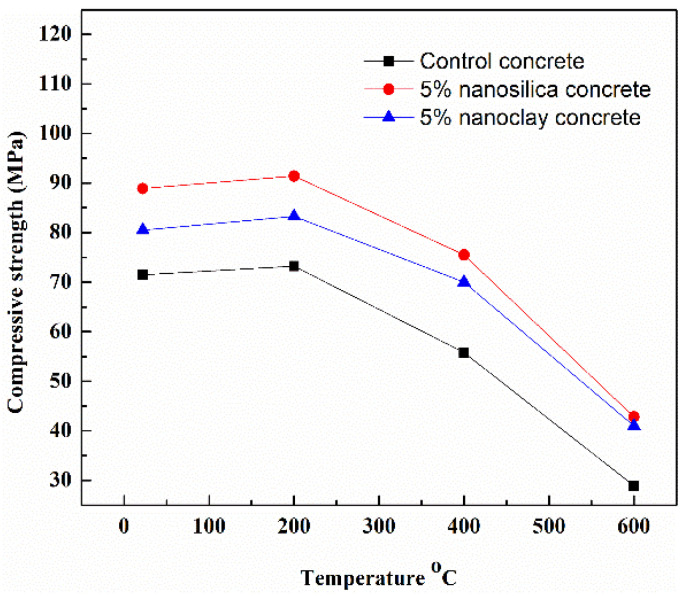
Compressive strength of control concrete, 5% nanosilica concrete, and 5% nanoclay concrete at 22, 200, 400, and 600 °C [80].

Figure 5 obviously highlights the impact of nanomaterials on enhancing compressive strength at elevated temperatures [80]. In Figure 5, the effect of nanomaterial enhancement on residual compressive strength is obvious when comparing the results to control concrete. It was reported that silica fume was added to the control sample [80], but the concrete with nanosilica and nanoclay exhibited higher compressive strength than the plain concrete under high temperatures, indicating that nanomaterials can provide concrete with better thermal properties than supplementary cementitious materials such as silica fume.

**Figure 6:**
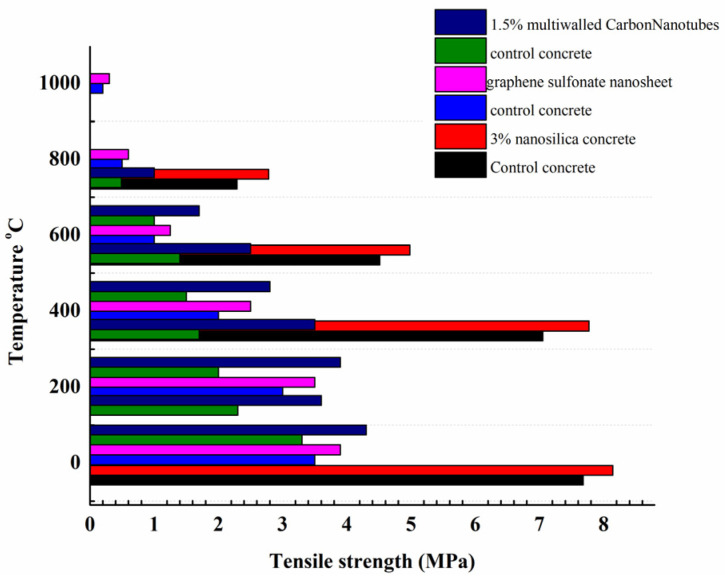
Tensile strength of concrete under different temperatures [71,74,77].

**Figure 7:**
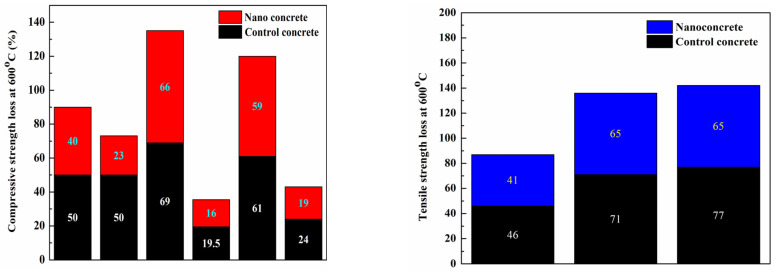
Compressive and tensile strength loss of concrete at 600 °C [71,73,74,75,76,77].

According to Figure 6, the results for concrete tensile strength at high temperatures [71,74,77] showed the same trend toward decreasing compressive strength, demonstrating the influence of temperature on tensile strength. The concrete’s tensile strength was decreased after raising the temperature, which was identical to the findings for its compressive strength. However, the incorporation of nanomaterials such as nanosilica or multi-walled nanotubes reduced the thermal damage caused by heat, thus increasing the residual tensile strength. Figure 7 shows the compressive and tensile strength losses of concrete at 600 °C.

Brzozowski et al. [68] reported that the thermal resistance enhancement with nanosilica was attributed to its thermal characteristics as well as the cement paste porosity structure when they used a method that was good at dispersing the nanoparticles in concrete. It was reported that the 0.3–300 m diameter pores were reduced with nanosilica particles, which also limited the microcracks and strengthened the ITZ zone. It was shown that the utilization of nanosilica in concrete exhibited significant enhancements in its mechanical properties, even at elevated temperatures of 600 °C. The rationale behind this improvement was attributed to the dense formation of calcium silicate hydrate (C-S-H), which is characterised by high rigidity. In a study by Nikbin et al. [75], the application of titanium dioxide as a nanomaterial was also investigated for its impact on the shielding performance of heavy concrete. In order to measure the effect of high temperatures on concrete strength, compressive strength tests were conducted on samples exposed to various levels of heat, including 25, 200, 400, and 600 °C. According to the findings, the compressive strength of concrete at high temperatures was enhanced with the incorporation of titanium dioxide. Pachideh et al. [77] also investigated the influence of carbon nanotubes on post-heat-treated concrete by adding different amounts of carbon nanotubes (0.5, 1, and 1.5 wt.%) to concrete and exposing the samples to temperatures of 25 °C, 100 °C, 250 °C, 500 °C, and 700 °C using an electric furnace. The study showed that the compressive strength of concrete increased by 10% at 25 °C, 16% at 100 °C, 39% at 250 °C, 11% at 500 °C, and 76% at 700 °C when a 0.5% dosage of carbon nanotubes was used. Furthermore, with a 1.5% dosage of carbon nanotubes, the compressive strength increased by 152% at 700 °C. The results highlight that the strong bonding between the cement paste and aggregates is what makes carbon nanotubes particularly effective at increasing the compressive strength of concrete subjected to heat. Additionally, carbon nanotube-modified concrete also demonstrated an increase in tensile strength at elevated temperatures. According to the findings of the study, incorporating carbon nanotubes in concrete can significantly enhance its tensile strength, particularly at higher temperatures. The study examined the effect of carbon nanotubes on the modulus of elasticity of concrete at different elevated temperatures and found that it increased up to 1.33 times at 25 °C, 1.1 times at 100 °C, 1.12 times at 250 °C, 1.45 times at 500 °C, and 1.84 times at 700 °C compared to control concrete. This suggests that carbon nanotubes can effectively improve the performance of concrete under high-temperature conditions.

Another study conducted by Kumar et al. [70] demonstrated that nanosilica high-strength concrete experienced an increase in compressive and split-tensile strength when exposed to temperatures up to 400 °C for 2 h. In comparison with conventional high-strength concrete, nanosilica high-strength concrete displayed higher residual compressive and split-tensile strength during exposure to temperatures up to 800 °C for 2 h. Interestingly, nanosilica high-strength concrete showed brittle failure only up to 600 °C, whereas conventional high-strength concrete exhibited brittle failure only up to 400 °C. Therefore, incorporating nanosilica into concrete can provide superior resistance to high-temperature environments and prevent premature failure in the structure. It was noted that conventional high-strength concrete had better ductility behaviour than nanosilica high-strength concrete. Wang [73] discovered that nanoclay concrete had greater strength when the temperature was maintained below 300 °C. At temperatures varying from 440 to 580 °C, the compressive strength was significantly reduced. The nanoclay concrete showed a 10% decrease in strength at 1000 °C when compared to the original concrete. The study indicated that nanoclay increased the compressive strength at 0.3 wt.% and 0.5 wt.% by weight dosages.

In a study by Mohammed et al. [79], the impact of combining graphene oxide with normal and high-strength concrete was examined under high temperatures of 800 °C. The results showed that the mechanical characteristics of concrete were improved due to the addition of graphene oxide, as evidenced by the residual compressive strength of 70% for the graphene oxide concrete compared to only 35% for the reference concrete. Chu et al. [74] assessed the mechanical characteristics of ferro-siliceous sacrificial concrete embedded with graphene sulfonate nanosheets during high-temperature exposure. According to the research conducted, the inclusion of 0.1 wt.% graphene sulfonate nanosheets was found to enhance the compressive strength and splitting tensile strength of concrete that was subjected to temperatures of up to 1000 °C. The reason for this improvement was believed to be due to the strengthening and toughening impact on the concrete’s microstructure, in addition to the decrease in overall porosity as the temperature increased. The conclusion drawn from this study was that graphene sulfonate nanosheets can significantly enhance the high-temperature integrity of concrete.

In a study by Nikbin et al. [75], the effect of different temperatures (25 °C, 200 °C, 400 °C, and 600 °C) on heavy-weight concrete incorporated with varying amounts of nano-bismuth oxide (Nano-Bi_2_O_3_) was analysed. Tests were conducted on the compressive strength of concrete exposed to high temperatures, and it was found that an increase in the content of nano-bismuth oxide led to a linear increase in the compressive strength. The study also revealed that there was an increase in the compressive strength for all samples as the temperature rose to 200 °C and 400 °C. However, at 600 °C, the compressive strength decreased, with the highest compressive strength seen in the samples containing 6 wt.% nano-bismuth oxide. In general, the study concluded that adding 6% of nano-bismuth oxide particles was the optimum content to enhance the mechanical properties of concrete exposed to high temperatures.

In their study, Sakthieswaran et al. [78] evaluated the behaviour of concrete modified with nanoalumina and containing zircon sand as fine aggregate when exposed to different elevated temperatures. To examine the performance of the concrete mixture, various tests were conducted on concrete samples exposed to temperatures ranging from 200 °C to 800 °C. The study found that the mechanical properties of the concrete improved due to the nanofiller effect of the nanoalumina, which reduced the pores and strengthened the interfacial transition zones in the concrete. The study also revealed that an increase in the percentage of nanoalumina in the concrete mixture resulted in higher compressive strength. In addition, the modified concrete exhibited greater flexural strength than the control concrete at all temperatures tested. These findings suggest that adding nanoalumina to the concrete mixture improves both its compressive and flexural strength, particularly at high temperatures. Furthermore, compared to the control concrete, the nanoalumina-modified concrete had considerably lower capillary porosity at normal and elevated temperatures. The decrease in porosity was attributable to the combined impact of zircon sand and the nano-alumina pore-filling capacity.

Overall, these findings suggest that incorporating nanoalumina into concrete is a promising approach for enhancing the mechanical properties of concrete, particularly when it is expected to be exposed to elevated temperatures.

### 3.3. Mass Loss and Spalling Improvements with Nanomaterials

Bastami et al. [71] conducted a study on the effects of heat on high-strength concrete that was blended with nanosilica, specifically looking at its mass loss and spalling tendencies. Concrete cracking and spalling were visibly observed at temperatures of 600 °C, and the decomposition of the aggregate occurred as the temperature reached 800 °C. The temperature at which nanosilica concrete experienced spalling and mass loss was greater than 400 °C, while the temperature at which ordinary concrete experienced spalling and mass loss was greater than 300 °C. At 600 and 800 °C temperatures, concrete spalling ranged from insignificant (a pitting surface) to severe (an explosive surface). The impact of nanosilica on spalling was not given much emphasis in the published work, as it mainly focused on its ability to decrease concrete permeability and enhance built-in tension stresses.

Shah et al. [72] performed a study showcasing how high-strength concrete’s properties were influenced by the presence of both nano- and microsilica when subjected to high temperatures. The results showed better spalling resistance in nanosilica specimens but higher temperature-induced cracking. It was observed by Mohammed et al. [79] that graphene oxide concrete has better fire-induced spalling resistance due to the reinforcing mechanism of graphene oxide and its ability to create networks of microchannels, facilitating the release of vapour pressure.

Table 2 lists the results for mass loss in concrete modified with nanomaterials at high temperatures.

### 3.4. Microstructure Observation of C-S-H Gel with Nanomaterials at High Temperatures

Increasing the temperature (i.e., less than 200 °C) can help make the concrete microstructure denser and more homogenous because it accelerates the cement hydration and increases the formed C-S-H gel stiffness. However, microcracks can be formed in plain concrete at temperatures higher than 400 °C, while temperatures above 800 °C cause the decomposition of cement gel. The incorporation of nanomaterials assists in providing a denser and more compacted microstructure even at higher temperatures, such as 400 °C. The improvement can be attributed to the nanoparticle effect on forming high-density C-S-H gel [70,75]. High-density C-S-H gel has higher thermal stability, which helps mitigate heat damage. Nanomaterials help create more arranged clusters of C-S-H gel in concrete when subjected to high temperatures. Several SEM images revealed that the concrete microstructure incorporated with nanomaterials at high temperatures had fewer cracks and cavities as compared to the control sample. Nanomaterials enhance the bond strength between cement paste and aggregates, which helps improve concrete’s resistance to heat. XRD images showed that nanoconcrete had a lower CH peak and a higher intensity of C-S-H at 200–400 °C, indicating the formation of an additional content of cement gel in the presence of nanoparticles.

## 4. Acid Resistance of Concrete Enhanced with Nanomaterials

The sustained attack of acid on concrete can severely harm and degrade its microstructure, ultimately resulting in material destruction [83]. The concrete comprising industrial construction such as sewer pipes, wastewater treatment plants, silos, dairies, and cooling towers is prone to acid attack, and the damage is extremely severe when the concrete surface is not protected. The acidity level affects how intense an acid attack is, and concrete’s significant damage occurs when the pH value of the acid solution is lower than 4.5 [84]. The three deterioration mechanisms of acidolysis, complexolysis, and decalcification were used to describe how acid solutions cause concrete to deteriorate [85]. The severity of an acid attack [86,87] is conditioned by several variables, such as concrete permeability, cement alkalinity, the content of calcium hydroxide in the cement matrix, the pH values or concentrations of acid solutions, and the solubility of formed salts. Based on their aggressiveness level, acid solutions can be classified into high-aggressive acids (e.g., hydrochloric, acetic, nitric, and sulfuric acids) and low-aggressive acids (e.g., phosphoric and humic acids). The most common acids encountered by concrete are sulfuric acid, nitric acid, hydrochloric acid, and carbonic acid [88]. The degradation mechanism underlying an acid attack on cementitious materials is mainly associated with the decalcification process of cement hydrates. Decalcification is the process of removing calcium and hydroxide ions from cement hydrates during exposure to acid solutions. The decalcification process causes the dissolution of the hydrated phases of cement paste. Based on hydrate solubility, CH dissolves first, followed by the hydrated phases with a greater Ca/Si ratio. At slightly lower pH values, the AFm and AFt phases typically dissolve. When calcium hydroxide is completely consumed, the dissolution of the C-S-H gel begins. The Ca/Si ratio affects the rate of C-S-H dissolution as well. In the most severe cases of acid attack, the dissolution of calcium–silicate–hydrate gel can seriously harm concrete’s structural integrity.

Certain techniques and strategies can be used to modify concrete’s resistance to an acid attack, such as concrete surface coating, altering the environment to make it less hostile to the concrete, adding fibres, and enhancing the impermeability of concrete. Incorporating nanomaterials helps increase the mechanical properties of concrete and its durability. Therefore, nanomaterials offer a wide range of applications for improving concrete’s resistance to an acid attack.

The effect of nanosilica and nanometakaolin on the resistance of high-strength and high-performance concrete against an acid attack was studied by Diab et al. [89]. The study focused on the analysis of the effect of various nanomaterials on concrete’s porosity, compressive strength, and water capillary absorption, as well as the impact of an acid attack on the material’s expansion strain, mass, and ultrasonic pulse velocity (UPV). The findings demonstrated that nanomaterials significantly reduced concrete’s porosity and improved its microstructure, ultimately enhancing its acid resistance. Moreover, nanomaterials reduced the water absorption percentage due to the nanofiller effect and porosity refinement mechanism. The bonding strength of the cement paste and aggregate was improved with nanomaterial incorporation. The higher the added content of nanomaterials, the lower the water absorption of concrete. The study revealed that nanomaterials can efficiently reduce the compressive strength loss of concrete caused by acid attacks. Furthermore, the results demonstrated that nanomaterials considerably reduced the concrete mass loss and UPV loss. Using scanning electron microscopic images, the microstructures of nanomaterial concrete were found to exhibit a lower amount of CH, gypsum, and ettringite needle crystals, in addition to a denser and more homogenous structure. The expansion strain that was caused by a nitric acid attack was greatly reduced with the incorporation of nanomaterials in concrete. In general, the stronger concrete exhibited less expansion after an acid attack; therefore, the compressive strength enhancement provided by nanomaterials proved to have better concrete expansion resistance. Due to nanomaterials’ pozzolanic activity that reduces the hydroxyl ion content and forms a higher amount of calcium silicate gel, which seals the capillary pores and increases concrete’s impermeability, less permeable concrete exhibits lower expansion under an acid attack.

In a study by Mahdikhani et al. [90], concrete’s mechanical properties and durability were investigated using the inclusion of nanosilica at varying concentrations of 0–6% and exposure to acid rain environments. The study evaluated mass loss, compressive strength, electrical resistivity, and water absorption of concrete under conditions of acid rain with different pH levels. To create acid rain, the researchers utilised sulfuric acid salt at a pre-determined pH level. The findings suggested that incorporating nanosilica improved concrete’s ability to resist acidic environments. Additionally, increasing the pH level of the acid rain resulted in enhanced mechanical properties and durability of the concrete. Various pH values were used to test the compressive strength of concrete containing nanosilica. With increasing the nanosilica amount in concrete at a higher pH value, the compressive strength was obviously increased. The destruction of concrete was observed in solutions with a lower pH value of 2.5, while at higher pH values of 7, 5.5, and 4.4, the damage caused by the acid attack was negligible. Sujay et al. [91] conducted experimental research to assess the durability of composite fibre-reinforced high-performance concrete incorporating nanosilica and ultra-fine fly ash. The concrete was immersed in solutions containing 5% concentrations of hydrochloric acid, sulfuric acid, and magnesium sulphate. Weight loss, residual compressive strength, and compressive strength were tested for concrete subjected to acid attack. With increasing the acid immersion period, the weight loss of concrete was increased. Sulfuric (H_2_SO_4_) acid immersion had the greatest weight loss effect on concrete, while hydrochloric (HCl) acid immersion had the least effect when only minimal weight loss was observed. This can be attributed to sulfuric acid’s combined damage from sulphate attack and acid attack. Compared to control concrete, nanomodified concrete had a lower weight loss. According to the study, the decline in the concrete’s residual compressive strength increased with the duration of acid soaking. Acids reduced the residual compressive strength of concrete by increasing the formation of ettringite in the concrete’s microscopic structure as the duration of acid soaking increased. However, adding a higher amount of nanosilica and ultra-fine fly ash worked to reduce the loss in residual compressive strength of concrete during its exposure to HCl and H_2_SO_4_. Decreasing residual strength loss was a result of the packing effect of nanomaterials, resulting in a dense microstructure and a reduction in capillary pores due to the higher amount of cement gel that improves the concrete’s resistance against acid attacks.

Praveenkumar et al. [92] studied the effects of a HCl attack on the strength and durability properties of concrete with TiO_2_ nanoparticles and rice husk ash. The study revealed that the longer the duration of acid immersion, the greater the weight loss of the concrete. Furthermore, the control concrete exhibited more damage than the concrete with nanomaterials due to acid exposure. The combination of 10% rice husk ash and different nano-TiO_2_ contents improved the concrete’s resistance to the HCl acid deterioration effect. The results indicated that 3 wt.% nano-TiO_2_ was the best dosage to improve acid resistance in concrete. Cao et al. [93] evaluated the effect of multi-walled carbon nanotubes on cementitious material resistance to sulfuric acid. The study used the diffusion–dissolution–precipitation mechanism in its explanation for acid degradation. Using their observation of the microstructure, it was concluded that gypsum formation was the main degradation effect through the sulfuric acid attack, owing to gypsum’s ability to provide pathways for ions to invade the materials, thus creating a damage cycle. The effect of multi-walled carbon nanotubes on enhancing sulfuric acid corrosion resistance was attributed to their role in mitigating ion diffusion by compacting the microstructure, promoting the hydration process, and forming a protective barrier in acid conditions. The inclusion of multi-walled carbon nanotubes was found to regulate the onset of microcracks and impede the spread of flaws such as cracks and detrimental pores. Furthermore, after incorporating multi-walled carbon nanotubes, the permeability and resistance to cracking of the cement matrix were enhanced. At the same time, the microstructure improvement induced with nanoparticles mitigated the concrete volume change that caused by gypsum formation and weakened expansion pressure. Table 3 summarises the results of studies that examined the impact of utilising nanomaterials on the acid resistance of concrete.

**Figure 8:**
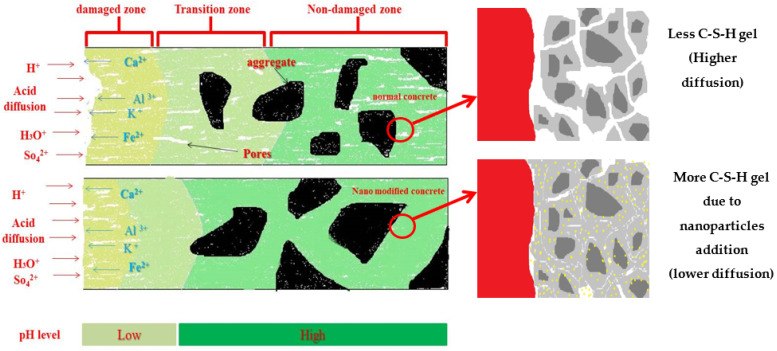
Schematic illustration showing the damage zones caused by an acid attack between plain concrete and nanomodified concrete.

Figure 8 is a schematic drawing demonstrating the damage zones caused by an acid attack between plain concrete and nanomodified concrete. It can be seen from the figure that the nanoparticles have a positive effect by compacting the microstructure, thereby decreasing the effect of acid invasion and reducing the depth of the damage zone compared to plain concrete.

## 5. Sulphate Resistance of Concrete Enhanced with Nanomaterials

A sulphate attack [94,95] has a dramatic deterioration effect on concrete through the sulphate reaction with cement hydrates, producing new solid phases at a relatively large volume. These new reaction products in the hardened cementitious matrix exert tensile stresses due to crystallisation pressure. Once these tensile stresses locally suppress the tensile strength of the material, cracks would develop, compromising the structure of the concrete. A sulphate attack can occur either as an external attack or as an internal attack. An external sulphate attack is characterised by sulphate contained in the surrounding environment (e.g., soils, groundwater, seawater, etc.) that infiltrates through the pores in the concrete and reacts with compounds in the cement paste such as CH, C-S-H gel, and monosulphate. An internal sulphate attack occurs when there is an excessive amount of sulphate in the raw materials of concrete, such as cement, aggregates, water, mineral and chemical admixtures, etc. The attack of sulphate on concrete can be characterised by chemical or physical degradation. An external chemical attack happens with the penetration of sulphate-containing solutions into the pores in the concrete, which increases the concentration of sulphate ions (SO4^2−^) that react with the source of calcium (Ca^2+^) and alumina to form ettringite (CaO·Al_2_O_3_·3CaSO_4_·32H_2_O) and with the source of calcium to form gypsum (CaSO_4_·2H_2_O). Ettringite is an expansive component, and its formation in hardened concrete could result in expansion, cracking, and mass loss, particularly when restrained. The formation of gypsum results in the softening of the concrete microstructure and loss of cohesion. A chemical attack by sulphate can involve the destabilisation of C-S-H gel and CH, generating microcracks within the concrete structure. A physical salt attack by sulphate can happen when concrete is in contact with soil. The groundwater contains deleterious ions, mostly sulphate, that infiltrate through concrete pores either by capillary sorption or diffusion. When the water inside the pores is evaporated by the dry air, salt will crystallise in the pores near the surface of the concrete. Salt precipitation and crystal growth exert crystallisation pressure, and whenever that pressure is greater than the tensile strength of the pore wall in which the crystals grow, surface scaling can occur, such as that caused by cycles of freezing and thawing. The most commonly occurring and naturally occurring sulphates that attack concrete are calcium (CaSO_4_·2H_2_O), sodium (NaSO_4_), and magnesium sulphate (MgSO_4_). The main three forms of damage caused by these salts are (1) the formation of ettringite and gypsum that cause expansion and strength loss; (2) the salt’s physical attack; and (3) thaumasite formation, which causes C-S-H destabilisation.

The denser microstructure and refined pore structure due to nanomaterial incorporation improve the permeability-related properties of concrete. Many studies revealed that nanomaterial addition to concrete had a huge impact on reducing concrete’s water absorption, providing denser microstructure in the interfacial transition zone (ITZ), and reducing pore connectivity [27,96,97]. Thus, the incorporation of nanomaterials can significantly improve concrete’s resistance to sulphate attack and reduce the deterioration effect of sulphate on its mechanical properties.

**Figure 9:**
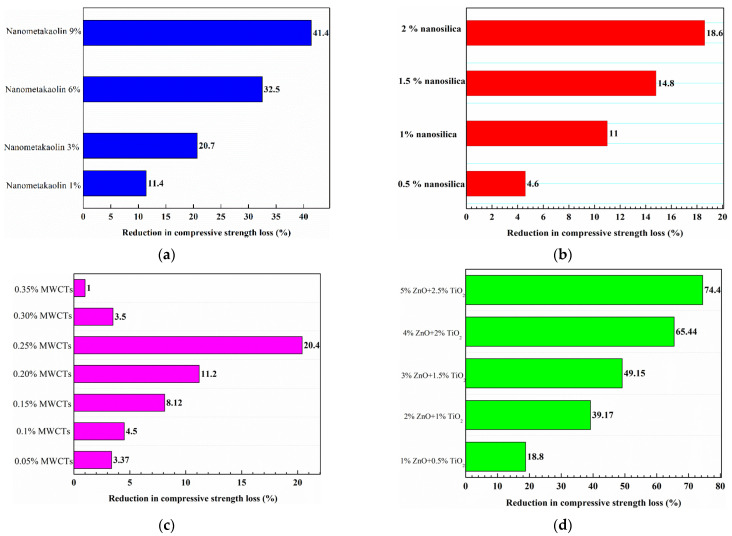
The reduction in concrete compressive strength loss (%) due to a sulphate attack after the addition of nanomaterials (**a**) The effect of different contents of nanometakaolin on reducing compressive strength loss of concrete subjected to 360 days of magnesium sulphate [89]. (**b**) The effect of different contents of nanosilica on reducing compressive strength loss of concrete subjected to 360 days of magnesium sulphate [89]. (**c**) The effect of different contents of multi-walled carbon nanotubes (MWCNTs) on reducing compressive strength loss of concrete subjected to 90 days of sodium sulphate [98]. (**d**) The effect of different contents of ZnO + TiO_2_ on reducing compressive strength loss of concrete subjected to 28 days of a sulphate solution [99].

Figure 9 demonstrates the effect of nanomaterials on the compressive strength loss of concrete due to a sulphate attack, as found in different studies. In Figure 9a,b, it is shown that the addition of nanosilica and nanometakaolin to high-strength concrete (55 MPa) subjected to 360 days of immersion in a 10% magnesium sulphate solution can linearly increase the enhancement in residual compressive strength [66]. The higher the added dosage, the higher the reduction in compressive strength loss. Magnesium sulphate has a considerable level of aggressiveness due to its combined effect of forming an expansive compound such as gypsum and destabilising cement hydrates such as CH and C-S-H gel by producing magnesium hydroxide. At a later stage of attack, magnesium sulphate reacts with C-S-H gel to form gypsum, magnesium hydroxide (MgOH_2_), and silica gel. The MgOH_2_ lowers the pH of the pore solution and causes decalcification of the C-S-H gel by exchanging calcium ions with magnesium ions, forming a weak non-cementitious substance known as magnesium silicate hydrate. Therefore, the destructive effect of a magnesium sulphate attack is associated with both cracking and softening. The longer the immersion duration of concrete in a magnesium solution, the higher the loss in compressive strength. However, the study showed that nanomaterials (nanosilica and nanometakaolin) enhanced the strength of concrete against sulphate attack due to their filling action and pozzolanic reactivity, which contribute to porosity reduction and compacting the microstructure. Multi-walled carbon nanotubes (MWCNTs) [98] improved the compressive strength loss of concrete due to a sulphate attack after 90 days of exposure. In the study, the concrete specimens with MWCNTs were immersed in a sodium solution for 28, 50, and 90 days. Like the previous study, the results indicated that the higher the exposure to sodium sulphate attack, the greater the reduction that can be induced in concrete’s compressive strength. It was observed that the reduction in concrete’s compressive strength loss increased with the addition of MWCNTs in all immersion periods until a certain limit of addition. The results of the study revealed that the specimens with a 0.25% addition of MWCNTs can be regarded as having the optimum content for minimising the compressive strength loss of the concrete. In a study by Reshma et al. [99], the impact of zinc oxide (ZnO) and titanium dioxide (TiO_2_) on the mechanical properties of concrete was assessed both with and without polypropylene fibres. The results presented in Figure 9d [99] illustrate the positive effects of incorporating ZnO and TiO_2_ on the residual compressive strength of non-fibrous concrete. The data showed a linear relationship between the amount of nanomaterial added and the reduction in compressive strength loss of the concrete. The improvements in compressive strength loss were due to the ability of ZnO and TiO_2_ to enhance C-S-H gel formation and increase the bond between aggregate and cement in the concrete.

**Figure 10:**
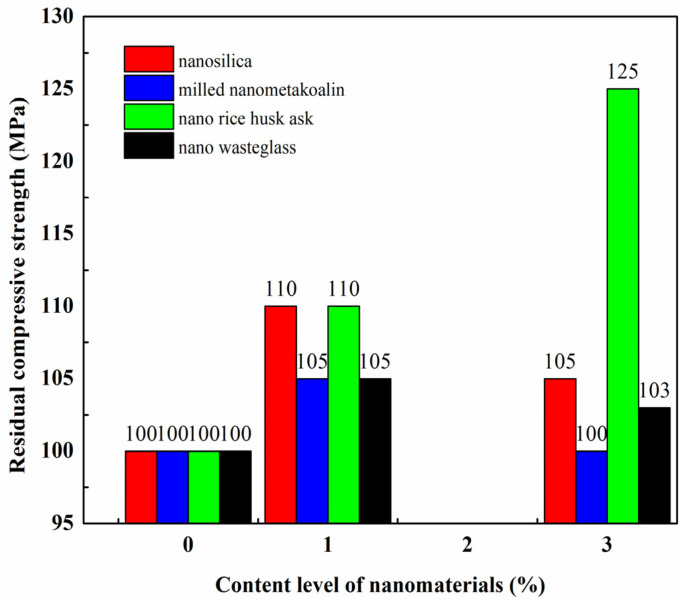
Residual compressive strength of air-cured, nanomodified concrete subjected to a sulphate solution [51].

Faried et al. [51] performed a study to investigate how the mechanical strength and durability of ultra-high-performance concrete were affected by varying curing conditions. To achieve the study’s aim, four different nanowaste materials—milled nanometakaolin, nanowaste glass, nanorice husk ash, and chemically prepared nanosilica—were added to the ultra-high-performance concrete. The study measured the residual compressive strength of several samples of the modified concrete after exposing them to a concentrated solution of sodium sulphate and magnesium sulphate for a period of 28 days. The research findings indicated that all the concrete specimens experienced reduced compressive strength due to a sulphate attack. However, the residual compressive strength of the nanomaterial-modified concrete was considerably higher compared to the unmodified concrete. Figure 10 presents the results for the residual compressive strength of the air-cured, nanomodified concrete that was exposed to the sulphate solution [51].

Several studies were also conducted to assess the weight loss of nanomaterial-modified concrete as a result of a sulphate attack [89,98,99,100,101]. Table 4 shows the data collected from various studies, which indicated that prolonged exposure to a sulphate solution increased the weight loss for all concrete samples. However, the use of nanomaterials significantly contributed to reducing the weight loss of the modified concrete.

**Figure 11:**
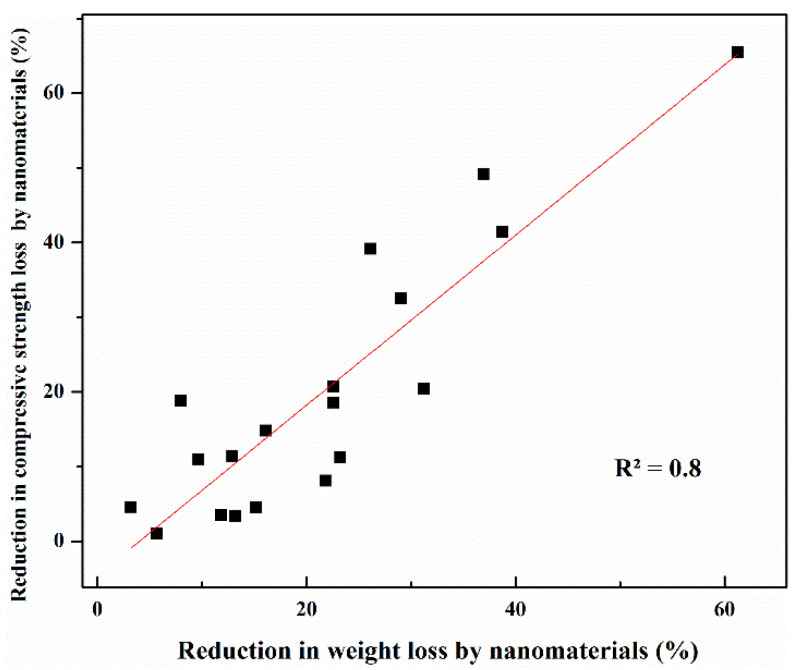
Correlation between the reduction in compressive strength loss and reduction in weight loss for concrete with nanomaterials [89,98,99,100,101].

Figure 11 depicts how nanomaterials enhanced the performance of concrete by reducing weight loss and compressive strength loss using a relationship that was developed between the mass loss and residual compressive strength improvement based on the data from several studies [89,98,99,100,101]. The fitting curve shows a good correlation between the two parameters. The higher the residual compressive strength of concrete exposed to sulphate attack, the lower the weight loss.

However, a study by Vishwakarma et al. [102] reported that when subjected to ammonium and sodium sulphate solutions, concrete structures containing nanoparticles were shown to be less resistant to sulphate attack than fly ash-modified concrete. Three different types of concrete were subjected to 3% ammonium and sodium sulphate for 90 days. The test results for these three types of concrete indicated a significant weight loss for specimens incorporated with fly ash, 2 wt.% nano-TiO_2_, and nano-CaCO_3_ compared to fly ash concrete without nanomaterial incorporation when the concrete specimens were subjected to 3% ammonium sulphate. Nano-TiO_2_-fly ash concrete exhibited higher weight loss than fly ash concrete. However, concrete with fly ash and nano-CaCO_3_ had the lowest weight loss percentage among other types of concrete. For concrete specimens subjected to a 3% sodium sulphate solution, the nano-TiO_2_-fly ash concrete had the highest percentage of weight loss, followed by fly ash-nano-CaCO_3_ concrete. Table 5 lists the weight loss for concrete with different types of nanomaterials. The fly ash concrete type had the lowest weight loss percentage among the other types. The researchers reached the conclusion that fly ash concrete treated with nanoparticles is not suitable for a sulphate-rich environment and that fly ash is better for sulphate attack resistance. This conclusion was attributed to the fact that fly ash decreases the percentage of C_3_A components, reducing the risk of sulphate attack by reducing the potential for formatting ettringite and gypsum.

The promotion of C-S-H gel formation with the pore refinement effect has been found to have a noteworthy correlation with the improvement in concrete’s mechanical properties and durability when added with nanomaterials. Solid evidence and explanation revealed that the total capillary porosity of concrete is decreased with nanomaterial incorporation due to the nanoparticle filler mechanism and high surface area; moreover, nanoparticles can act as nucleation sites for additional hydrate formation, increasing the C-S-H gel content in the cement matrix. External sulphate ingress through the concrete is controlled by permeability (porosity-dependent) when the phase composition and pore network determine the rate of sulphate ion penetration. In addition, the amount of water absorbed is directly related to the level of porosity in the concrete. Due to their super fineness, nanoparticles, such as nanosilica, have proven to be highly efficient in decreasing the water absorption into concrete. The nanosilica’s ability to densify and improve the microstructure of concrete by filling the porous hydration products and reacting with CH to produce more gel hydrates would block water transport channels and inhibit the diffusion of aggressive ions. On the other hand, Wang et al. [103] found that the content level of nanosilica is a crucial factor for water absorption; when the content level exceeded 5%, it had an adverse impact on material resistance to sulphate penetration and concentration. The reason for this adverse impact was attributed to the nanosilica particle agglomeration effect, which has a negative impact on hydration phases and material structure compactness. The research also showed that adding nanosilica to concrete decreased the SO_4_^2−^ apparent diffusion coefficients; however, an excessive nanosilica incorporation had a larger deviation rate of the apparent diffusion coefficients. Reshma et al. [99] investigated the impact of nanomaterials such as ZnO and TiO_2_ on the water absorption and sorptivity of concrete and reported a beneficial outcome. It is clear that the addition of nanomaterials to a cement mixture can enhance the durability of concrete, as it improves its microstructure by filling gaps and increasing pozzolanic reactivity, thus resisting sulphate attacks.

## 6. Chloride Ion Penetration of Concrete Enhanced with Nanomaterials

Reinforced concrete structures are very vulnerable to chloride ion ingress, which can result in steel corrosion [104]. When steel bars corrode due to chloride-induced corrosion, it weakens the structure’s strength and safety by causing spalling and reducing the steel cross-sectional area. Therefore, it is critical and urgent to safeguard concrete against chloride ion corrosion [105]. The permeability of materials, the capacity of chloride binding, and the ion exchange capacity all greatly impact the diffusion of chloride ions [106] into concrete.

The addition of nanoparticles to concrete helps to enhance its mechanical characteristics, including compressive strength, by elevating the quantity of C-S-H gel and optimising the structure of the concrete’s pores. This is achieved when the particles react with CH to form a denser microstructure, thus enhancing material durability. Multiple experiments and explanations have confirmed that nanomaterials can improve concrete’s resistance to chloride attacks.

Singh et al. [107] reported that the incorporation of nanosilica significantly improved resistance to chloride ion diffusion. Their study comparing the effectiveness of nanosilica and silica fume in mitigating chlorine attacks found that adding 3% nanosilica increased resistance to chloride ions by as much as 43%, whereas silica fume only improved it by 15%. The enhancement in nanosilica’s ion penetration resistance was attributed to its ability to promote the cement hydration process and create a denser microstructure as compared to silica fume. This assumption was supported by monitoring the cement microstructure development of both nanosilica and silica fume using SEM, TGA, and compressive strength. An examination was carried out by Rezakhani et al. [108] to investigate the effects of water pressure and rebar corrosion on chloride ion (Cl^−^) diffusion in graphene oxide-modified concrete. Various types of concrete were subjected to constant water pressures of 0.3, 0.5, and 0.7 MPa for a duration of 144 h to infiltrate chloride ions. The outcome of the research showed that the rate of chloride penetration was increased by both the water pressure and the depth of immersion in concrete, regardless of the type of concrete. However, the graphene oxide–slag-based, cement-modified concrete displayed improved resistance against ion penetration. The positive impact of nanomaterials on this type of concrete was observed at higher pressure, which caused a significant reduction in the measured diffusion coefficients by a factor of 3. The nanofiller effect of graphene oxide was responsible for blocking the pores and cutting the ions’ diffusion paths in concrete. However, the higher content level of graphene oxide might be detrimental due to the agglomeration effect of concrete, highlighting the importance of caution regarding the use of nanomaterials.

**Figure 12:**
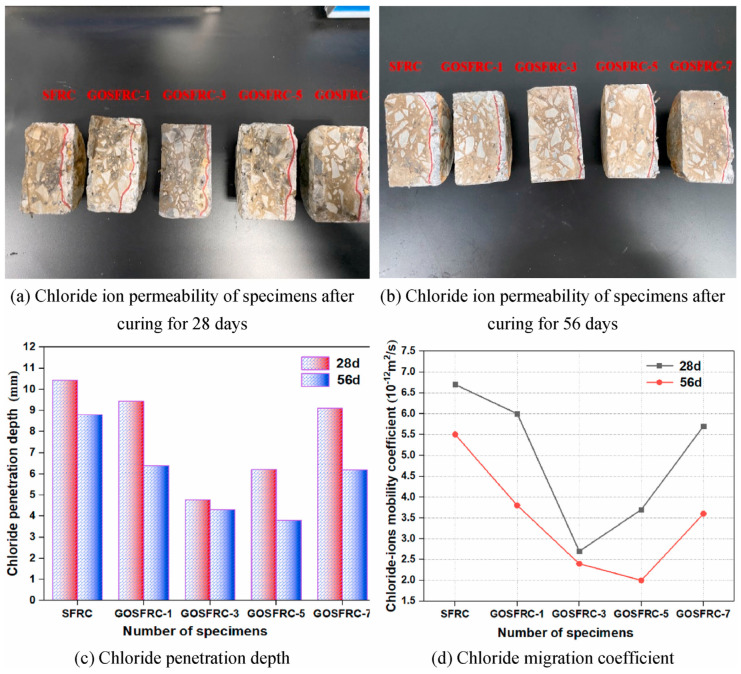
The chloride ion resistance of graphene oxide-modified, steel fibre-reinforced concrete. Reproduced with permission from [109], (*Cement and Concrete Composites*); published by (Elsevier), (2022) (Note: SFRC: 0% graphene oxide, GOSFR-1: 0.01%wt. graphene oxide, GOSFRC-3: 0.03 wt.%, GOSFRC-5: 0.05 wt.%, GOSFRC: 0.07% graphene oxide).

Liu et al. [109] investigated the chloride ion migration coefficient for steel fibre-reinforced concrete treated with graphene oxide. Their findings revealed that resistance to chloride ion ingress into concrete was significantly enhanced by increasing the curing time and incorporating graphene oxide. The experimental results demonstrated that 0.03 wt.% and 0.05 wt.% of graphene oxide significantly reduced the chloride ion penetration depth and lowered the chloride migration coefficient. Moreover, 0.03 wt.% of graphene oxide was found to be the optimum amount for improving the concrete’s resistance to chloride. The chloride ion resistance of graphene oxide-modified, steel fibre-reinforced concrete is illustrated in Figure 12 [109].

**Figure 13:**
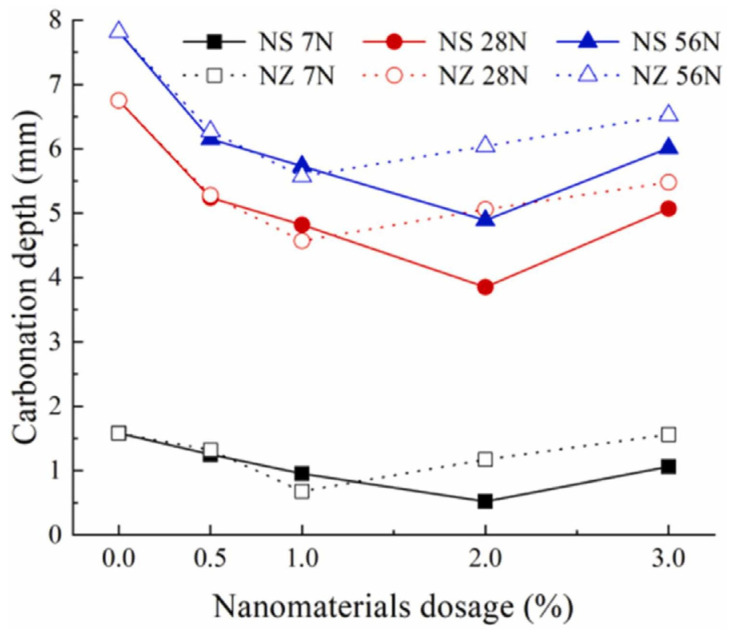
Free Cl^−^ concentration in nanoconcrete at different nanomaterial dosages (0–5 mm). Reproduced with permission from [110], (*Case Studies in Construction Materials*)*;* published by (Elsevier), (2023) (Note: NS: NanoSiO_2_, NZ: Nano-ZnO, N = number of cycles).

Zhang et al. [110] evaluated the durability of marine concrete doped with nanoparticles. The concentration of free chlorides and carbonation depth of the concrete were regarded as the durability index. Nanosilica and nano-ZnO were used, and the test was performed by measuring the chloride content and carbonation depth after immersing the concrete in a 5% NaCl solution for a total of 56 cycles. The concentration of free chlorides was higher at lower depths and decreased with increasing depth, indicating that the Cl^−^ ions accumulated closer to the concrete surface. Moreover, it can be observed from Figure 13 [110] that the free concentration of Cl^−^ ions in nanoconcrete was reduced compared to that of plain concrete. This suggests that the addition of nanomaterials enhanced the concrete’s ability to resist penetration by chloride ions. However, the 2% addition of nanosilica was more effective at increasing the concrete’s resistance to chlorides compared to the 1% nano-ZnO concrete. The carbonation depth of nanoconcrete is lower compared to plain concrete. The optimal performance of concrete regarding anti-carbonation and chloride resistance was achieved by incorporating the proper content of nanoparticles into the concrete, and adding 1% of nano-ZnO and 2% of nanosilica was noted to be the optimum content levels. In contrast, the concrete’s chloride resistance and anti-carbonation performance were reduced by increasing the amount of nanomaterials. The adverse impact of exceeding the optimal content was due to the agglomeration effect and insufficient local cement hydration. Based on XRD test results, it was affirmed that nanosilica is more effective in consuming calcium hydroxide than nano-ZnO because nanosilica has smaller nanoparticles and thereby greater surface activity. By consuming the Ca(OH)_2_ phase, nanomaterials can drastically improve concrete’s resistance to chlorides due to the phase negative impact by reacting with Cl^−^ to form complexes that facilitate chloride diffusion and reacting with CO_2_ to form CaCO_3_, which reduces the concrete pH and accelerates steel corrosion. The incorporation of nanoparticles lowers the CaCO_3_ amount in concrete and produces a higher C-S-H gel content, thus enhancing the anti-carbonation performance of concrete under chlorine attack. On the other hand, nanosilica can enhance durability in a more functional way than nano-ZnO, due to the former’s advanced effect in promoting the formation of Friedel’s salts and improving the concrete microstructure.

**Figure 14:**
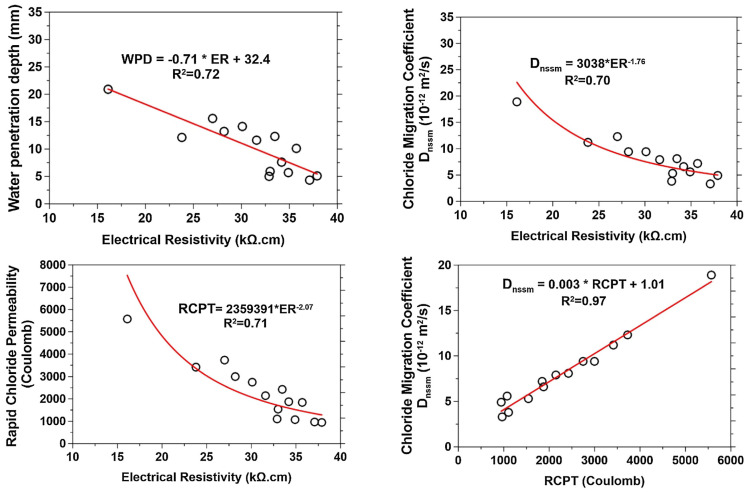
The relationship between electrical resistivity and durability parameters of nanoconcrete. Reproduced with permission from [111], (*Construction and Building Materials*); published by (Elsevier), (2020).

Joshaghani et al. [111] conducted a study on concrete that had been modified using different types of nanomaterials such as nano-TiO_2_, nano-Al_2_O_3_, and nano-Fe_2_O_3_. Their research covered areas such as water penetration and electrical resistivity, as well as the rapid chloride permeability test and the rapid chloride migration test. The study emphasised that nanomaterial-modified concrete had lower permeability and lower water penetration than plain concrete due to the nanoparticles’ role in reducing the pore volume and connectivity. A lower permeability indicates lower chemical attacks due to the inhibition of the ingress of aggressive ions such as chlorides into concrete. The 91-day water penetration depths of 0.32 w/c ratio concrete incorporated with 5% TiO_2_, Al_2_O_3_, and Fe_2_O_3_ were found to be 51%, 59%, and 64% lower than the control concrete, respectively. The improvement in water penetration with nanomaterial incorporation was interpreted as the cause of nanoparticle dilution and heterogeneous nucleation effects. Promoting cement hydration through pozzolanic reactivity with nanoparticle addition was expected to result in a higher amount of cement gel, which would reduce the pores connectivity and hinder the water transport within concrete. The electrical resistivity of concrete surfaces increased over time as the hydration process continued. This increase in the solid phase blocks electrical currents. The nanomodified concrete had a greater electrical resistivity than control concrete due to the porosity-refining effect of nanomaterials. The electrical response of concrete is a useful indicator of permeability. A linear correlation between water penetration and surface resistivity tests was established. An increase in electrical resistivity led to a decrease in water penetration depth. Incorporating nanoparticles reduced the concrete’s penetrability, as confirmed using the rapid chloride migration tests. The chloride migration coefficient decreased significantly with the addition of nanoparticles. Electrical resistivity and chloride migration coefficients depended on the water-to-binder ratio and nanomaterial incorporation. These parameters were controlled by concrete porosity, pore size, and pore connectivity. The rapid chloride permeability tests showed decreased charge passing through concrete specimens when the w/b ratio was lowered and when nanoparticles were added. Nanoparticles improved concrete’s resistance to chloride penetration, especially with a higher w/b ratio, due to their porosity-refining effect. Nanotechnology helped to create durable concrete with low permeability and low chloride penetration rates. A linear relationship was found between electrical resistivity and the rapid chloride permeability of the concrete. Figure 14 demonstrates correlations between the electrical resistivity and durability parameters of nanoconcrete, which indicate that durability was strongly correlated to permeability. Thus, nanoparticle addition can enhance the microstructure and promote C-S-H gel content to improve concrete durability properties.

**Figure 15:**
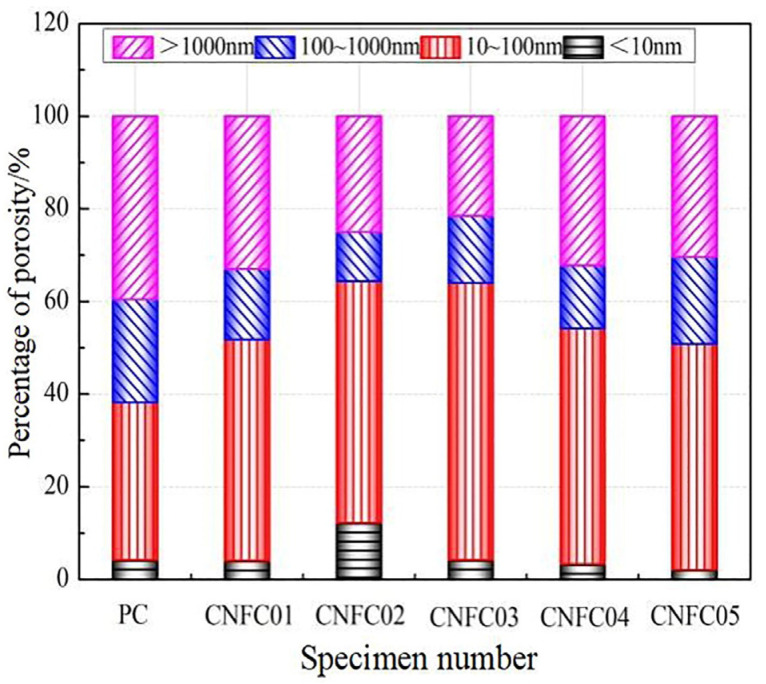
Percentage of porosity for carbon nanofiber concrete (CNFC) in different dosages (0.1–0.5%). Reproduced with permission from [112], (*Construction and Building Materials*); published by (Elsevier), (2020).

A rapid chloride permeability test was carried out by Sujay et al. [91] on high-performance concrete reinforced with composite fibres and treated with nanosilica and ultra-fine fly ash. It was deduced that the chloride ion permeability for composite fibre-reinforced, high-performance concrete with nanomaterials was significantly lower than plain concrete. Nanosilica acted by reducing the amount of charge passed within the concrete, and the effect was more clearly demonstrated with increasing the amount of nanosilica. It was found that the ideal replacement for concrete with nanosilica was 3%. The research by Wang et al. [112] indicated that the incorporation of carbon nanofiber into concrete, with a volume fraction ranging from 0.1 to 0.5%, resulted in a significant reduction in water seepage height and the relative permeability coefficient by 14.1%, 26.6%, 39.1%, 37.5%, and 32.8%, respectively, when compared to control concrete. In addition, the carbon nanofiber-modified concrete with a volume fraction of 0.3% had the lowest permeability. The improvement in permeability was mainly related to the pore structure in the concrete. Carbon nanofiber addition dramatically reduced the average pore size, median pore diameter, largest pore size, and total pore volume in the concrete. Figure 15 shows the porosity percentage for the carbon nanofiber-modified concrete [112], and it was observed that the percentage of macropores within carbon nanofiber-modified concrete was extremely lower than plain concrete. Nanomaterial incorporation increased the nanopores and micropores due to their high surface area and filler effect on reducing the size of macropores. Furthermore, the concrete containing 0.3% carbon nanofiber possessed the finest pore structure. When compared to ordinary concrete, adding 0.1 to 0.5% carbon nanofiber decreased the average size, median diameter, total volume of pores, and percentage of macropores by 58.9%, 81.9%, 99.6%, 16.4%, and 45.6%, respectively. However, the volume fraction of 0.5% had higher macropores than the optimum content of 3%, indicating that the pore structure of carbon nanofiber concrete can provide a better improvement. In all other respects, the maximum proportion of 0.5% had a better improvement over plain concrete. It was determined from the findings that the correct amount of carbon nanofibre should be added to the concrete to reduce capillary porosity and improve its microstructure.

Tests were carried out by Li [113] to evaluate the effects of nanosilica, nano-CaCO_3_, and multi-walled carbon nanotubes on the chloride permeability and chloride binding capacity of concrete. The study applied rapid chloride migration and equilibrium tests to quantify these properties. The results indicated that the inclusion of 2 wt.% nanosilica proved to be extremely effective at decreasing the chloride diffusion coefficient of the concrete, with the coefficient of nanosilica-enhanced concrete being 85.3% lower than that of plain concrete. The improvement using nanosilica was attributed to the nano-filler effect and its role in blocking the harmful pores and microcracking inside the concrete matrix, intensifying the microstructure, and hindering chloride transport. Furthermore, nanosilica promoted the hydration process with a nucleation effect that leads to the formation of higher amounts of gel-like hydrates, which increase the strength of cement paste and their bond to aggregate. Reducing the CH crystals with the pozzolanic activity of nanosilica was associated with improving the concrete’s strength by forming a denser C-S-H gel and strengthening the interfacial transition zone. These three effects of nanosilica (the nanofiller effect, nucleation effect, and pozzolanic effect) decreased the pore volume and subsequently lowered chloride diffusion. However, at higher incorporated amounts of nanosilica, such as 4 wt.%, the concrete resistance was less compared to 2 wt.%. The adverse impact was attributed to agglomeration and poor dispersion of nanoparticles owing to the van der Waals force, which causes weak zones and defects in the cement matrix. Regarding the nano-CaCO_3_ incorporation, the effect was also significant in improving the concrete resistance to chlorides by reducing the diffusion coefficient with a low-dosage addition, due to the nanofiller effect and nucleation effect as well as the chemical effect. The addition of 4 wt.% of nano-CaCO_3_ had a negative impact on chloride diffusion as compared to plain concrete. The negative impact was attributed to the agglomeration effect of nanoparticles. For the multi-walled carbon nanotube addition, the results showed remarkable improvement in regard to chloride resistance by reducing the chloride diffusion coefficient. The improvement resulted from the chemical effect of absorbing the Ca^2+^ from the pore solution, promoting the crystallisation and production of hydration products, in addition to the nucleation and bridging effects of bonding the C-S-H and improving the microstructure. The agglomeration effect was also present with a 4 wt.% addition, which reduced the effectiveness of nanomaterial enhancement of chloride resistance. Mercury intrusion porosimetry (MIP) was conducted to link the pore characteristics with concrete resistance to chlorides. The results showed that nanomaterial incorporation reduced the total pore volume of concrete. The content of smaller-diameter pores was increased with nanomaterial incorporation, which was considered harmless. The results indicated a refinement in pore structure with nanomaterials. Small pores are characterised as isolated local pores, while large pores are often connected as interconnected networks in concrete. Nanomaterials, due to their filler effect, turn the large pores into small pores, increasing the tortuosity of the system and hindering chloride transport in concrete. The diffusion coefficient was increased by increasing the capillary pores in the concrete. The increased concrete diffusion caused by increasing porosity was attributed to an increase in connected pores. Chloride binding can mitigate the chloride attack and reduce rebar corrosion in concrete by removing the chloride ions. However, the incorporation of nanomaterials compromises the chloride binding in concrete due to reducing the amount of CH by the pozzolanic reaction and reducing the Ca/Si ratio of C-S-H. By reducing the amount of CH, the pH in concrete would be reduced, the alkalinity would be lowered, and this would lower the chloride binding capacity.

Recent studies by Lu et al. [49] and Liu et al. [50] focused on the effect of nanomaterial incorporation on the durability of recycled aggregate concrete. The investigation also included testing the water sorptivity and chloride ion permeability. A graphene oxide–cement slurry was used to coat the recycled aggregates with the purpose of strengthening the interface transition zone (ITZ) microstructure [49]. The results revealed that the graphene oxide coating helped reduce the water absorption coefficient by 15.1%, improved the microstructure, reduced the microcracks, reduced the ITZ by 44.4%, lowered the total charge passed by 63.4%, and significantly mitigated the chloride ion penetration of concrete. The impermeability, chloride ion resistance, and freezing–thawing resistance were investigated for recycled coarse aggregate concrete modified by coating the coarse aggregate with nanosilica and sodium silicate [50]. Regarding the concrete, the coating technique utilizing nanoparticles and coarse aggregates yielded increased mechanical strength, superior durability characteristics, and reduced pore content. Nanosilica has widespread use not only because of its lower cost compared to other nanomaterials but also because of its filling, nucleation, and pozzolanic properties.

## 7. Surface Abrasion Resistance of Concrete Optimised with Nanomaterials

**Figure 16:**
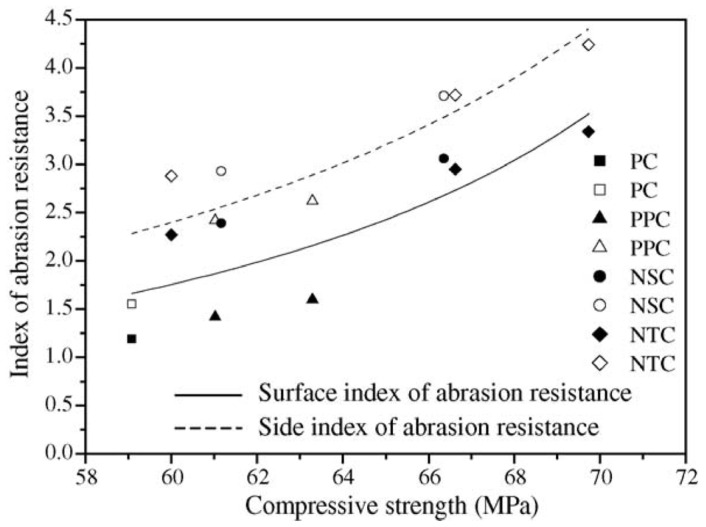
The relationship between the index of abrasion resistance and compressive strength for all mixtures of concrete. Reproduced with permission from [117], (*Wear*); published by (Elsevier), (2006) (Note: PC: control concrete, PPC: polypropylene fibre concrete, NSC: nanosilica concrete, NTC: nano-TiO_2_ concrete).

Concrete durability mainly depends on the abrasion resistance property. Abrasion resistance defines how surfaces resist rubbing and friction [114]. This characteristic is crucial for pavements subjected to continuous dynamic loads from traffic. The strength, porosity, and aggregate properties of concrete greatly influence its ability to resist abrasion [115]. Concrete with low compressive strength is susceptible to rapid abrasion, as it affects its surface integrity. A previous published work showed a linear correlation between compressive strength and abrasion resistance [116]. Incorporating nanomaterials into concrete significantly improved its mechanical properties, and therefore its abrasion resistance. Li et al. [117] demonstrated that the addition of nano-TiO_2_ and nanosilica into concrete enhanced its abrasion resistance compared to concrete containing polypropylene fibres. The effect of nanomaterial incorporation was best observed with 1 wt.% nanosilica or nano-TiO_2_, which had an index increment of up to 157% and 180%, respectively. These enhancements were due to the nanomaterials’ ability to control crystallisation in cement paste, prompting the formation of more C-S-H gel, and leading to a compacted and homogenous cement matrix. However, the amount of nanoparticles added should be optimum, as higher amounts result in less improvement. A linear relationship between abrasion resistance and compressive strength was identified for plain concrete, concrete with fibres, and concrete with nanomaterials as shown in Figure 16. Compressive strength is a primary factor influencing the abrasion resistance of concrete.

**Figure 17:**
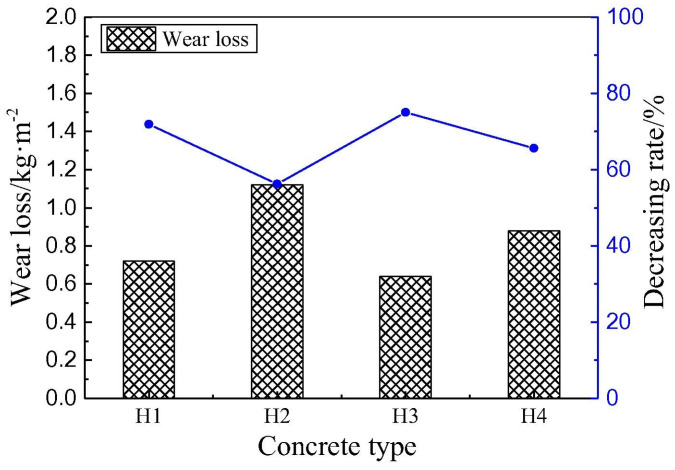
The results of wear resistance for nano-double-doped concrete. Reproduced with permission from [52], (*Construction and Building Materials*); published by (Elsevier), (2017) (Note: H1: 1% nanoSiO_2_ + 3% nanoSIC, H2: 3% nanosilica + 1% nanoSIC, H3: 2% nanoSiO_2_ + 2% nanoSIC, H4: 2% nanoSiO_2_ + 1% nanoSIC).

**Figure 18:**
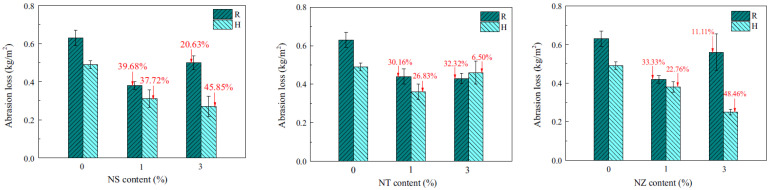
The abrasion loss of reactive powder concrete containing nanomaterials. Reproduced with permission from [118], (*Construction and Building Materials*); published by (Elsevier), (2018). (Notes: R: cured at room temperature, H: heat curing, NS: nano-SiO_2_, NT: nano-TiO_2_, NZ: nano-ZrO_2_).

Gao et al. [52] investigated the wear resistance of fly ash concrete that underwent modification using nano-silica or nanoSiC. The results of the study revealed that the incorporation of nanomaterials significantly enhanced the wear resistance of fly ash concrete when compared to reference concrete. Furthermore, the analysis showed that the wear loss in the presence of nanosilica and nano-SiC was remarkably reduced. However, it was discovered that increasing the concentration of nanoparticles led to a linear decrease in the wear loss, and consequently, the optimal content of nanomaterial was determined to be 2 wt.%. To visually represent the wear loss in concrete incorporated with the two types of nanomaterials, Figure 17 was presented. The outcomes of the investigation indicated that the effect of wear resistance was better on nano-double-doped concrete as opposed to that of nano-single-doped concrete. Of all the concrete types tested, H3 concrete displayed the best effect. The findings also revealed that H1 had superior performance concerning wear loss compared to H2, suggesting that nano-SiC had a more notable impact on wear resistance. In summary, the study highlighted the significant improvement of wear resistance in fly ash concrete with the addition of nanomaterials, most notably nano-silica and nanoSiC. Wang et al. [118] examined the enhancement caused by nanoparticles on the wear resistance of reactive powder concrete. Three types of nanomaterials were used for the investigations: nanosilica, nano-TiO_2_, and nano-ZrO_2_. Figure 18 shows the abrasion loss for concrete with different nanomaterial content using two curing approaches: ambient curing and heat curing.

**Figure 19:**
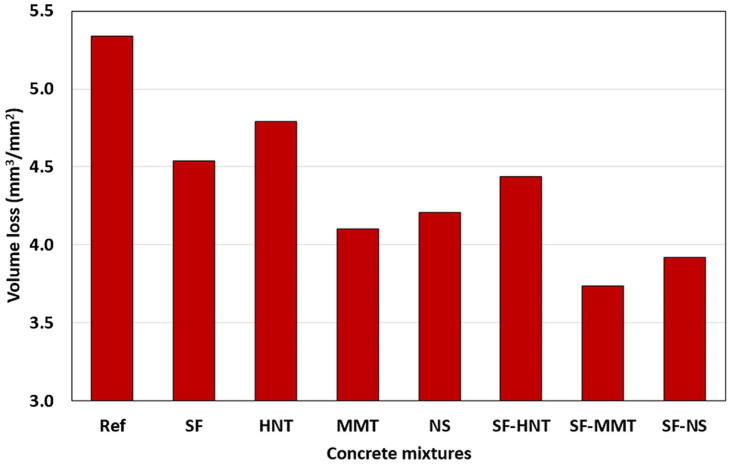
The loss of concrete volume per surface area after 16 periods of wearing. Ref: control concrete, SF: silica fume concrete, HNT: nano-halloysite MMT: montmorillonite, NS: nanosilica. Reproduced with permission from [119], (*Construction and Building Materials*); published by (Elsevier), (2020).

From Figure 18, it can be observed that all types of nanomaterials improved the abrasion resistance of reactive powder concrete. However, better abrasion resistance was observed for reactive powder concrete containing nanomaterials cured at elevated temperatures, except for 3 wt.% nano-TiO_2_. The nanomaterial content level for the three concretes was between 1 and 3 wt.%. For room temperature curing, 1 wt.% content was the optimum dosage for the three types of nanomaterials regarding abrasion loss. Nanosilica reactive powder concrete had the best enhancement effect on abrasion loss, whether using room temperature curing or at heat curing, when the enhancement rate reached 39.68% for a 1 wt.% dosage and 37.72% for a 3 wt.% dosage. In ascending order, the wear resistance of room temperature-cured reactive powder concrete was improved by nanoparticles, namely nanoZrO_2_-3%, nanosilica-3%, nanoTiO_2_-1%, nanoTiO_2_-3%, nanoZrO_2_-1%, and nanosilica-1%. For heat-cured reactive powder concrete, the improvement increased in the order of nanoTiO_2_-3%, nanoZrO_2_-1%, nanoTiO_2_-1%, nanosilica-1%, nanosilica-3%, and nanoZrO_2_-3%. The improvement in wear resistance with nanomaterials was attributed to their effect on enhancing the interfacial transit zone and structure of cement. The results of the study elucidated that increasing the content of nanoparticles decreased the concrete’s resistance to abrasion due to an increasing trend in CH crystal orientation. Microstructural analysis and surface characteristics of nanoparticle-containing concrete pavements were studied by Ghoddousi et al. [119]. The abrasion resistance was tested for concrete containing nanomontmorillonite, nanosilica, and nanohalloysite. Similar to the above-mentioned results, the study revealed that nanomaterials profoundly improved the concrete pavement wear resistance when wear loss was remarkably lower than that of the reference concrete. Figure 19 depicts the loss of concrete volume per surface area after 16 wear cycles. The results showed that nanomaterial-modified concrete had less volume loss as compared to silica fume concrete and reference concrete. The improvement of 3 wt.% nano-montmorillonite, 2 wt.% nano-silica, and 3 wt.% nano-halloysite to the wear resistance of concrete was 23.2%, 21.2%, and 10.3%, respectively. For concrete with 10 wt.% silica fume, the improvement was 15%. The reason for the lower improvement was due to the higher replacement level of silica fume, increasing the content of cementitious composite and reducing the presence of aggregate, which greatly contributed to concrete abrasion resistance. However, the blending silica fume and nanomaterials together into concrete can profoundly increase the material’s wear resistance. The greatest improvement was observed in the result for the silica fume-nanomontmorillonite-modified concrete sample. It was believed that the enhancement brought about using silica fume and nanoparticles had a more important effect on strengthening the abrasion resistance of the concrete samples than the reduction in the presence of aggregates that occurred as a result of SCM addition.

**Figure 20:**
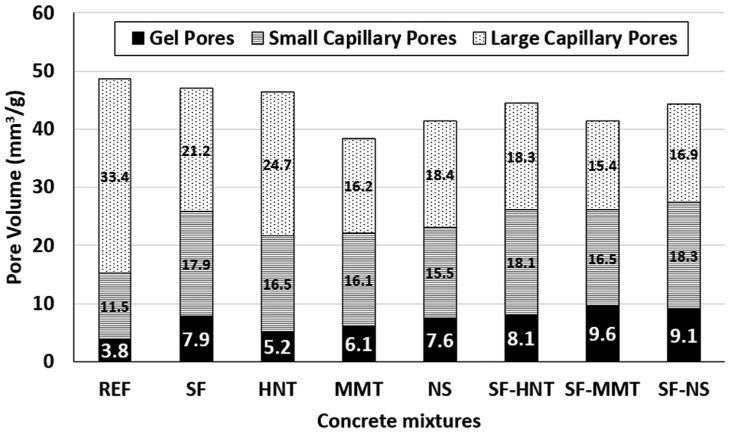
The pore volume of nanoconcrete. REF: control concrete, SF: silica fume concrete, HNT: nano-halloysite MMT: montmorillonite, NS: nanosilica. Reproduced with permission from [119], (*Construction and Building Materials*); published by (Elsevier), (2020).

To evaluate how porosity and the pore size distribution impact abrasion resistance, concrete samples underwent Mercury intrusion porosimetry (MIP) testing. Figure 20 presents the volume of small and large capillary and gel pores across all concrete types. Among these, the silica fume and nanomaterials concrete sample had a reduced content of large capillary pores, resulting in higher resistance against abrasion. Comparatively, the nano-halloysite concrete exhibited greater volume loss and a higher content of large capillary porosity. The study found that nano-halloysite had less effect on converting large porosity into small capillary pores and gel pores when compared to nano-montmorillonite and nanosilica. As a result, the total porosity of nano-halloysite increased by 21.1% and was 14.8% less than that of nano-montmorillonite and nanosilica, respectively. Silica fume concrete showed only a 3.5% reduction in pore volume when compared with reference concrete. Therefore, the impact of silica fume on concrete porosity was considered insignificant compared to the effects of nanomaterials. The weight loss was correlated with the compressive strength and average pore diameter of the concrete. The study revealed that a higher compressive strength leads to lower volume loss due to abrasion, while a larger average pore diameter results in higher weight loss.

Wang et al. [120] performed pore structural and fractal analysis on the abrasion resistance and cracking resistance of concrete. It was discovered that concrete porosity and pore surface fractal dimensions measured using MIP were linearly correlated to the concrete abrasion resistance. In addition, the dimensional intricacy or unevenness of a material’s pores, known as pore surface fractal dimensions, has a greater impact on the abrasion resistance of concrete than its porosity. The study revealed that both concrete strength and abrasion resistance increased with decreasing porosity and increasing pore surface fractal dimensions. As such, using ultrafine materials such as nanoparticles to refine the pore structure and diameter can greatly increase concrete’s surface abrasion resistance and mechanical strength.

NanoMgO is another nanomaterial used to reduce the surface cracking of concrete caused by autogenous shrinkage and thermal stresses [121]. On the other hand, microMgO [122] functions as an agent that limits concrete cracking caused by thermal shrinkage in mass concrete and self-desiccation from utilizing a low w/c ratio and fine material. However, Wang et al. [123] reported that excessive amounts of microMgO can deteriorate compressive strength, tensile strength, and abrasion resistance in concrete. Therefore, nanoMgO is a better alternative for reducing shrinkage and improving compressive strength of concrete simultaneously. A few studies [124,125,126] indicated that nanoMgO is more effective at reducing autogenous shrinkage compared to microMgO while also enhancing the mechanical properties and permeability of cementitious materials by developing a more uniform and compact microstructure. Adding 1% nanoMgO to concrete reduced permeability by up to 63% and improved other mechanical properties such as compressive and tensile strength, according to Yazdchi et al. [124]. Ye et al. [127] conducted SEM and XRD investigations to examine the hydration and microstructure effects of nanoMgO on cementitious material and found that it improved its microstructure.

## 8. Conclusions

This article concentrates on enhancing the hydration of C-S-H gel while examining the robustness of nanoconcrete. Incorporating nanomaterials into concrete reduces porosity and enhances its microstructure by increasing the content of cement gel. The mechanical strength and permeability of concrete are improved by the nanofiller, pozzolanic, and nucleation effects of nanoparticles. In addition, the nanoparticle effect reduces concrete capillary porosity and total pore volume by promoting the hydration of unhydrated cement particles and pozzolanic reactions with CH crystals. By increasing the amount of C-S-H gel produced with the nanomaterial reaction, the material’s resistance to external attack by sulphates or acids is significantly increased, and the permeability and water absorption of concrete are reduced. The addition of nanomaterials such as nanosilica, nanoclay, and graphene oxide was proven to enhance concrete’s durability against high temperatures as well as acid or sulphate degradation. Furthermore, nanoconcrete exhibits better performance against surface abrasion with the reduction in chloride penetration and wear load sustained. While an optimum amount of nanoparticles provides more durable and robust concrete, a higher amount could cause an adverse impact due to the agglomeration effect. To enhance the performance of nanoconcrete, recommendations include conducting more studies on its durability and resistance to other types of threats, broadening the types of nanomaterials added, and exploring better techniques and approaches for better nanoparticle dispersion in concrete.

## Figures and Tables

**Table 1:** A summary of the reviews on the influence of various nanomaterials on concrete’s resistance to heat.

Reference	Nanomaterial	Subjected Temperatures	Highlights
Brzozowski et al. [68]	Nanosilica	200, 400, 600, 800 °C	Nanosilica improved the residual strength and reduced the pore percentage, having a size of 0.3 to 300 μm.
Kumar et al. [69]	Nanosilica	200, 400, 600, 800 °C	Nanosilica reduced the thermal conductivity, mass loss, and the concrete elastic modules, and the residual strength increased.
Bastami et al. [70]	Nanosilica	400, 600, 800 °C	Nanosilica reduced the concrete mass loss, while residual strength increased.
Shah et al. [71]	Nanosilica	200, 500, 800 °C	Nanosilica concrete showed more significant cracks than microsilica concrete at 800 °C, but nanosilica concrete spalled less than micro- + nanosilica concrete.
Wang et al. [72]	Nanoclay	25–1000 °C	Nanoclay improved the residual strength; 0.1 wt.% nanoclay reduced the thermal conductivity coefficient of cement.
Chu et al. [73]	Graphene sulfonate nanosheets	Up to 1000 °C	Graphene sulfonate nanosheets increased compressive strength, splitting tensile strength, and thermal diffusivity. Porosity was reduced.
Nikbin et al. [74]	Nano bismuth oxide	200, 400, 600 °C	Nano-bismuth oxide particles with higher amounts reduce concrete weight loss.
Nikbin et al. [75]	Titanium dioxide	200, 400, 600 °C	Better performance regarding compressive strength was observed due to titanium dioxide incorporation.
Pachideh et al. [76]	Carbon nanotubes	100, 250, 500, 700 °C	Reduced the risk of fire-induced spalling.
Sakthirswaran et al. [77]	Nanoalumina	200, 400, 600, 800 °C	Nanoalumina particles strengthened the IZT zone and reduced porosity.
Mohammed et al. [78]	Graphene oxide	400, 600, 800 °C	The compressive strength of normal and high-strength concretes exposed to heat increased with Graphene oxide addition.

**Table 2:** Mass loss (%) of concrete modified with nanomaterials under different temperatures.

Reference	Concrete Admixture	200 °C	400 °C	500 °C	600 °C	800 °C	1000 °C
Bastami et al. [71]	0 wt.% nanosilica		4.13		9.26	18.56	
	1.5 wt.% nanosilica		4.03		7.16	14.26	
	3 wt.% nanosilica		3.38		7.24	10.82	
	4.5 wt.% nanosilica		3.20		8.48	11.76	
Shah et al. [72]	10 wt.% microsilica	2.50		5.00		15.00	
	5 wt.% nanosilica	3.00		7.00		10.00	
	10 wt.% microsilica + 5 wt.% nanosilica	3.00		10.00		20.00	
Chu et al. [74]	0 wt.% graphene sulfonate nanosheets	2.38	3.43		4.32	6.03	6.18
	0.1 wt.% graphene sulfonate nanosheets	2.12	3.08		4.04	5.42	5.61
Nikbin et al. [75]	0 wt.% Bi_2_O_3_	1.50	3.75		5.25		
	2 wt.% Bi_2_O_3_	1.00	3.10		5.30		
	4 wt.% Bi_2_O_3_	0.80	2.10		5.35		
	6 wt.% Bi_2_O_3_	0.8	2.00		5.25		
Nikbin et al. [76]	0 wt.% nanoTiO_2_	1.20	3.30		5.20		
	2 wt.% nanoTiO_2_	2.20	3.40		6.20		
	4 wt.% nanoTiO_2_	2.50	4.50		6.50		
	6 wt.% nanoTiO_2_	2.80	5.50		7.00		

**Table 3:** Summary of several studies that investigated the effect of using nanomaterials on the acid resistance of concrete.

Reference	Nanomaterials	Level Content (wt.%)	Acid Solutions	Key Findings	Discussion
Diab et al. [89]	Nanosilica	0.5%, 1%, 1.5%, 2%	Nitric acid, sulfuric acid	The effect of nitric acid was decreased with nanomaterial incorporation when the water capillary absorption, porosity, compressive strength loss caused by the acid attack, and expansion strains caused by the acid attack were reduced. The nanomaterial enhancement of concrete’s resistance to sulfuric acid was better than concrete’s resistance to nitric acid.	Microstructure improvement due to the filling effect of nanomaterials and pozzolanic activity.
	Nanometakaolin	1%, 3%, 6%, 9%			
Mahdikhani et al. [90]	Nanosilica	0%, 2%, 4%, 6%	Acid rain using sulfuric acid salt	Nanosilica improved the compressive strength of concrete in sulfuric conditions and the impermeability and durability of concrete.	Reduced porosity with the precipitation of higher hydrates due to changes in pore solution composition as well as an improvement in the bond strength between the matrix substances, owing to the filling impact of nanosilica.
Sujay et al. [91]	Nanosilica	15%	Hydrochloric acid, sulfuric acid	Lower weight loss with the addition of nanomaterials. In addition, the higher the content of nanosilica, the higher the residual compressive strength of concrete exposed to acids.	Microstructure improvement due to the filling effect of nanomaterials and pozzolanic activity.
	Ultra-fine fly ash	1.5%, 3%, 4.5%			
Praveenkumar et al. [92]	NanoTiO_2_	1%, 2%, 3%, 4%, 5%	Hydrochloric acid	Overall, 3% nanoTiO_2_ was the optimum amount for a lower mass loss of concrete exposed to hydrochloric acid. The combination of 10% rice husk ash and different nano-TiO_2_ amounts had greater resistance to deterioration when subjected to acidic conditions.	Improved the durability and strength of concrete.

**Table 4:** The weight loss for nanomaterial-modified concrete exposed to a sulphate attack.

Reference	Nanomaterials	Level of Content (%)	Sulphate Solution	Exposure Duration (Days)	Weight Loss (%)
Vijayabhaskar et al. [98]	MWCNTs	0	Na_2_SO_4_	90	2.11
	MWCNTs	0.05	Na_2_SO_4_	90	1.83
	MWCNTs	0.10	Na_2_SO_4_	90	1.79
	MWCNTs	0.15	Na_2_SO_4_	90	1.65
	MWCNTs	0.2	Na_2_SO_4_	90	1.62
	MWCNTs	0.25	Na_2_SO_4_	90	1.45
	MWCNTs	0.3	Na_2_SO_4_	90	1.86
	MWCNTs	0.35	Na_2_SO_4_	90	1.99
	MWCNTs	0.4	Na_2_SO_4_	90	3.01
Reshma et al. [99]	ZnO + TiO_2_	0	Sulphate solution	28	6.12
	ZnO + TiO_2_	1 + 0.5	Sulphate solution	28	5.63
	ZnO + TiO_2_	2 + 1	Sulphate solution	28	4.52
	ZnO +TiO_2_	3 + 1.5	Sulphate solution	28	3.86
	ZnO + TiO_2_	4 + 2	Sulphate solution	28	2.37
	ZnO + TiO_2_	5 + 2.5	Sulphate solution	28	2.21
Moslemi et al. [100]	NanoSiO_2_	0	Na_2_SO_4_	180	3.51
	NanoSiO_2_	2	Na_2_SO_4_	180	2.40
	NanoSiO_2_	4	Na_2_SO_4_	180	2.23
	NanoSiO_2_	6	Na_2_SO_4_	180	1.13
	NanoSiO_2_	8	Na_2_SO_4_	180	1.00
Diab et al. [89]	Nanometakaolin	0	MgSO_4_	360	3.10
	Nanometakaolin	1	MgSO_4_	360	2.70
	Nanometakaolin	3	MgSO_4_	360	2.40
	Nanometakaolin	6	MgSO_4_	360	2.20
	Nanometakaolin	9	MgSO_4_	360	1.90
	NanoSiO_2_	0	MgSO_4_	360	3.10
	NanoSiO_2_	0.5	MgSO_4_	360	3.00
	NanoSiO_2_	1	MgSO_4_	360	2.80
	NanoSiO_2_	1.5	MgSO_4_	360	2.60
	NanoSiO_2_	2	MgSO_4_	360	2.40
Sathe et al. [101]	NanoAl_2_O_3_	0	MgSO_4_	28	3.86
	NanoAl_2_O_3_	2.3	MgSO_4_	28	1.52

**Table 5:** Weight loss for concrete with different types of nanomaterials [102].

Concrete Type	Sulphate Solution	Weight Loss (%)
Fly ash concrete	Ammonium sulphate	1.8
Fly ash + Nano-TiO_2_ concrete	Ammonium sulphate	2.5
Fly ash + Nano-CaCO_3_ concrete	Ammonium sulphate	1.75
Fly ash + Nano-TiO_2_ + Nano-CaCO_3_ concrete	Ammonium sulphate	2.70
Fly ash concrete	Sodium sulphate	1.30
Fly ash + Nano-TiO_2_ concrete	Sodium sulphate	2.80
Fly ash + Nano-CaCO_3_ concrete	Sodium sulphate	2.20
Fly ash + Nano-TiO_2_ + Nano-CaCO_3_ concrete	Sodium sulphate	2.10

## Data Availability

No new data were created or analysed in this study. Data sharing is not applicable to this article.

## References

[1] Nazeer M., Kapoor K., Singh S.P. (2023). Strength, durability and microstructural investigations on pervious concrete made with fly ash and silica fume as supplementary cementitious materials. J. Build. Eng..

[2] Mehta P.K., Monteiro P.J.M. (2005). Durability in Concrete: Microstructure, Properties, and Materials.

[3] Al-Jabari M. (2022). Concrete durability problems: Physicochemical and transport mechanisms. Integral Waterproofing of Concrete Structures.

[4] Li X., Xu Q., Chen S. (2016). An experimental and numerical study on water permeability of concrete. Constr. Build. Mater..

[5] Cyr M. (2016). Influence of supplementary cementitious materials (SCMs) on concrete durability. Eco-Efficient Concrete.

[6] Pan X., Shi Z., Shi C., Ling T.C., Li N. (2017). A review on concrete surface treatment Part I: Types and mechanisms. Constr. Build. Mater..

[7] Isayed S.H., Amjad M.A. (1996). Strength, water absorption and porosity of concrete incorporating natural and crushed aggregate. J. King Saud Univ. Eng. Sci..

[8] Claisse P. (2021). Measurement of Porosity as a Predictor of the Transport Properties of Concrete in Transport Properties of Concrete: Modelling the Durability of Structures.

[9] Baghabra Al-Amoudi O.S., Al-Kutti W.A., Ahmad S., Maslehuddin M. (2014). Correlation between compressive strength and certain durability indices of plain and blended cement concretes. Cem. Concr. Compos..

[10] Peter C. (2017). Hewlett and Martin Liska. Lea’s Chemistry of Cement and Concrete.

[11] Goel G., Sachdeva P., Chaudhary A.K., Singh Y. (2022). The use of nanomaterials in concrete: A review. Mater. Today Proc..

[12] Reches Y. (2018). Nanoparticles as concrete additives: Review and perspectives. Constr. Build. Mater..

[13] Yoo D.Y., Oh T., Banthia N. (2022). Nanomaterials in ultra-high-performance concrete (UHPC)—A review. Cem. Concr. Compos..

[14] Silvestre J., Silvestre N., de Brito J. (2015). Review on concrete nanotechnology. Eur. J. Environ. Civ. Eng..

[15] Li L., Wang B., Hubler M.H. (2022). Carbon nanofibers (CNFs) dispersed in ultra-high performance concrete (UHPC): Mechanical property, workability and permeability investigation. Cem. Concr. Compos..

[16] Piro N.S., Salih A., Hamad S.M., Kurda R. (2021). Comprehensive multiscale techniques to estimate the compressive strength of concrete incorporated with carbon nanotubes at various curing times and mix proportions. J. Mater. Res. Technol..

[17] Abhilash P.P., Nayak D.K., Sangoju B., Kumar R., Kumar V. (2021). Effect of nano-silica in concrete; a review. Constr. Build. Mater..

[18] Liu R., Xiao H., Geng J., Du J., Liu M. (2020). Effect of nano-CaCO_3_ and nano-SiO_2_ on improving the properties of carbon fibre-reinforced concrete and their pore-structure models. Constr. Build. Mater..

[19] Ansari rad T., Tanzadeh J., Pourdada A. (2020). Laboratory evaluation of self-compacting fiber-reinforced concrete modified with hybrid of nanomaterials. Constr. Build. Mater..

[20] Ruan Y., Han B., Yu X., Zhang W., Wang D. (2018). Carbon nanotubes reinforced reactive powder concrete. Compos. Part A Appl. Sci. Manuf..

[21] Khalaf M.A., Cheah C.B., Ramli M., Ahmed N.M., Al-Shwaiter A. (2021). Effect of nano zinc oxide and silica on mechanical, fluid transport and radiation attenuation properties of steel furnace slag heavyweight concrete. Constr. Build. Mater..

[22] Maohua Z., Zhengyi L., Jiyin C., Zenong T., Zhiyi L. (2022). Durability of marine concretes with nanoparticles under combined action of bending load and salt spray erosion. Adv. Mater. Sci. Eng..

[23] Khooshechin M., Tanzadeh J. (2018). Experimental and mechanical performance of shotcrete made with nanomaterials and fiber reinforcement. Constr. Build. Mater..

[24] Ahmed T.I., El-Shafai N.M., El-Mehasseb I.M., Sharshir S.W., Tobbala D.E. (2022). Recent advances in the heating resistance, thermal gravimetric analysis, and microstructure of green concrete incorporating palm-leaf and cotton-stalk nanoparticles. J. Build. Eng..

[25] Yan J., Liu X., Wang X., Wang L., Weng W., Yu X., Xing G., Xie J., Lu C., Luo Y. (2022). Influence of nano-attapulgite on compressive strength and microstructure of recycled aggregate concrete. Cem. Concr. Compos..

[26] Narasimman K., Jassam T.M., Velayutham T., Yaseer M., Ruzaimah R. (2020). The synergic influence of carbon nanotube and nanosilica on the compressive strength of lightweight concrete. J. Build. Eng..

[27] Du S., Ge Y., Shi X. (2019). A targeted approach of employing nano-materials in high-volume fly ash concrete. Cem. Concr. Compos..

[28] Wang X., Ding S., Qiu L., Ashour A., Wang Y., Han B., Ou J. (2022). Improving bond of fiber-reinforced polymer bars with concrete through incorporating nanomaterials. Compos. B Eng..

[29] Jia H., Cui B., Niu G., Chen J., Yang Y., Wang Q., Tang C. (2023). Experimental and mechanism study on the impermeability and thermal insulation of foam concrete regulated by nano-silica and fluorine-free foam. J. Build. Eng..

[30] Shahpari M., Khaloo A., Rashidi A., Saberian M., Li J. (2023). Synergetic effects of hybrid nano-blended cement on mechanical properties of conventional concrete: Experimental and analytical evaluation. Structures.

[31] Meng T., Ying K., Yang X., Hong Y. (2021). Comparative study on mechanisms for improving mechanical properties and microstructure of cement paste modified by different types of nanomaterials. Nanotechnol. Rev..

[32] Wang Y., Lu H., Wang J., He H. (2020). Effects of Highly Crystalized Nano C-S-H Particles on Performances of Portland Cement Paste and Its Mechanism. Crystals.

[33] Liu C., He X., Deng X., Wu Y., Zheng Z., Liu J., Hui D. (2020). Application of nanomaterials in ultra-high performance concrete: A review. Nanotechnol. Rev..

[34] Wang B., Yao W., Stephan D. (2019). Preparation of calcium silicate hydrate seeds by means of mechanochemical method and its effect on the early hydration of cement. Adv. Mech. Eng..

[35] Land G., Stephan D. (2015). Controlling cement hydration with nanoparticles. Cem. Concr. Compos..

[36] Xue Q., Ni C., Wu Q., Yu Z., Shen X. (2021). Effects of Nano-CSH on the hydration process and mechanical property of cementitious materials. J. Sustain. Cem.-Based Mater..

[37] Hakamy A., Shaikh F., Low I. (2015). Characteristics of nanoclay and calcined nanoclay-cement nanocomposites. Compos. Part B Eng..

[38] Yuenyongsuwan J., Sinthupinyo S., O’Rear E.A., Pongprayoon T. (2019). Hydration accelerator and photocatalyst of nanotitanium dioxide synthesized via surfactant-assisted method in cement mortar. Cem. Concr. Compos..

[39] Khamchin F., Rasiah S., Sirivivatnanon V. Properties of Metakaolin Concrete—A Review. Proceedings of the International Conference on Sustainable Structural Concrete.

[40] Nazari A., Riahi S. (2010). The effects of ZrO_2_ nanoparticles on physical and mechanical properties of high strength self-compacting concrete. Mater. Res..

[41] Devi S.C., Khan R.A. (2020). Compressive strength and durability behavior of graphene oxide reinforced concrete composites containing recycled concrete aggregate. J. Build. Eng..

[42] Zhang P., Su J., Guo J., Hu S. (2023). Influence of carbon nanotube on properties of concrete: A review. Constr. Build. Mater..

[43] Wang J., Han B., Li Z., Yu X., Dong X. (2019). Effect Investigation of Nanofillers on C-S-H Gel Structure with Si NMR. J. Mater. Civ. Eng..

[44] Long G., Li Y., Ma C., Xie Y., Shi Y. (2018). Hydration kinetics of cement incorporating different nanoparticles at elevated temperatures. Thermochim. Acta.

[45] Sun Y., Zhang P., Guo W., Bao J., Qu C. (2020). Effect of Nano-CaCO_3_ on the mechanical properties and durability of concrete incorporating Fly Ash. Adv. Mater. Sci. Eng..

[46] Hussien R.M., Abd el-Hafez L., Mohamed R., Faried A.S., Fahmy N.G. (2022). Influence of nano waste materials on the mechanical properties, microstructure, and corrosion resistance of self-compacted concrete. Case Stud. Constr. Mater..

[47] Li G., Chen X., Zhang Y., Zhuang Z., Lv Y. (2023). Studies of nano-SiO_2_ and subsequent water curing on enhancing the frost resistance of autoclaved PHC pipe pile concrete. J. Build. Eng..

[48] Ren J., Lai Y., Gao J. (2018). Exploring the influence of SiO_2_ and TiO_2_ nanoparticles on the mechanical properties of concrete. Constr. Build. Mater..

[49] Lu D., Wang D., Wang Y., Zhong J. (2023). Nano-engineering the interfacial transition zone between recycled concrete aggregates and fresh paste with graphene oxide. Constr. Build. Mater..

[50] Liu X., Xie X., Liu R., Lyu K., Zuo J., Li S., Liu L., Shah S.P. (2023). Research on the durability of nano-SiO_2_ and sodium silicate co-modified recycled coarse aggregate (RCA) concrete. Constr. Build. Mater..

[51] Faried A.S., Mostafa S.A., Tayeh B.A., Tawfik T.A. (2021). Mechanical and durability properties of ultra-high performance concrete incorporated with various nano waste materials under different curing conditions. J. Build. Eng..

[52] Gao Y., He B., Li Y., Tang J., Qu L. (2017). Effects of nano-particles on improvement in wear resistance and drying shrinkage of road fly ash concrete. Constr. Build. Mater..

[53] Fahmy N.G., Hussien R.M., el-Hafez L.A., Mohamed R., Faried A.S. (2022). Comparative study on fresh, mechanical, microstructures properties and corrosion resistance of self compacted concrete incorporating nanoparticles extracted from industrial wastes under various curing conditions. J. Build. Eng..

[54] Asadi I., Shafigh P., Hassan Z.F.B.A., Mahyuddin N.B. (2018). Thermal conductivity of concrete—A review. J. Build. Eng..

[55] Saif M.S., Shanour A.S., Abdelaziz G.E., Elsayad H.I., Shaaban I.G., Tayeh B.A., Hammad M.S. (2023). Influence of blended powders on properties of ultra-high strength fibre reinforced self compacting concrete subjected to elevated temperatures. Case Stud. Constr. Mater..

[56] Ma Q., Guo R., Zhao Z., Lin Z., He K. (2015). Mechanical properties of concrete at high temperature—A review. Constr. Build. Mater..

[57] Almasaeid H.H., Suleiman A., Alawneh R. (2022). Assessment of high-temperature damaged concrete using non-destructive tests and artificial neural network modelling. Case Stud. Constr. Mater..

[58] Jiao Y., Liu H., Wang X., Zhang Y., Luo G., Gong Y. (2014). Temperature effect on mechanical properties and damage identification of concrete structure. Adv. Mater. Sci. Eng..

[59] Lin S., Wang Y., Suzuki Y. (2009). High-temperature CaO hydration/Ca(OH)_2_ decomposition over a multitude of cycles. Energy Fuels.

[60] Janotka I., Nürnbergerová T. (2005). Effect of temperature on structural quality of the cement paste and high-strength concrete with silica fume. Nucl. Eng. Des..

[61] Ali M.H., Dinkha Y.Z., Haido J.H. (2017). Mechanical properties and spalling at elevated temperature of high performance concrete made with reactive and waste inert powders. Eng. Sci. Technol..

[62] Mohammed H., Ahmed H., Kurda R., Alyousef R., Deifalla A.F. (2022). Heat-induced spalling of concrete: A review of the influencing factors and their importance to the phenomenon. Materials.

[63] Kannangara T., Joseph P., Fragomeni S., Guerrieri M. (2022). Existing theories of concrete spalling and test methods relating to moisture migration patterns upon exposure to elevated temperatures—A review. Case Stud. Constr. Mater..

[64] Zeiml M., Leithner D., Lackner R., Mang H.A. (2006). How do polypropylene fibers improve the spalling behavior of in-situ concrete?. Cem. Concr. Res..

[65] Bao J., Zheng R., Sun Y., Zhang P., Cui Y., Xue S., Song Q.A. (2023). state-of-the-art review on high temperature resistance of lightweight aggregate high-strength concrete. J. Build. Eng..

[66] Lu D., Tang Z., Zhang L., Zhou J., Gong Y., Tian Y., Zhong J. (2020). Effects of combined usage of supplementary cementitious materials on the thermal properties and microstructure of high-performance concrete at high temperatures. Materials.

[67] Sikora P., Abd Elrahman M., Stephan D. (2018). The influence of nanomaterials on the thermal resistance of cement-based composites—A review. Nanomaterials.

[68] Brzozowski P., Strzałkowski J., Rychtowski P., Wróbel R., Tryba B., Horszczaruk E. (2022). Effect of nano-SiO_2_ on the microstructure and mechanical properties of concrete under high temperature conditions. Materials.

[69] Kumar R., Singh S., Singh L.P. (2017). Studies on enhanced thermally stable high strength concrete incorporating silica nanoparticles. Constr. Build. Mater..

[70] Bastami M., Baghbadrani M., Aslani F. (2014). Performance of nano-silica modified high strength concrete at elevated temperatures. Constr. Build. Mater..

[71] Shah A.H., Sharma U.K., Roy D.A.B., Bhargava P. (2013). Spalling behaviour of nano SiO_2_ high strength concrete at elevated temperatures. MATEC Web of Conferences.

[72] Wang W.-C. (2017). Compressive strength and thermal conductivity of concrete with nanoclay under various high-temperatures. Constr. Build. Mater..

[73] Chu H., Jiang J., Sun W., Zhang M. (2017). Effects of graphene sulfonate nanosheets on mechanical and thermal properties of sacrificial concrete during high temperature exposure. Cem. Concr. Compos..

[74] Nikbin I.M., Rafiee A., Dezhampanah S., Mehdipour S.S., Mohebbi R., Moghadam H.H., Sadrmomtazi A. (2020). Effect of high temperature on the radiation shielding properties of cementitious composites containing nano-Bi_2_O_3_. J. Mater. Res. Technol..

[75] Nikbin I.M., Mehdipour S., Dezhampanah S., Mohammadi R., Mohebbi R., Moghadam H.H., Sadrmomtazi A. (2020). Effect of high temperature on mechanical and gamma ray shielding properties of concrete containing nano-TiO_2_. Radiat. Phys. Chem..

[76] Pachideh G., Gholhaki M., Moshtagh A., Felaverjani M.K. (2018). An Investigation on the effect of high temperatures on the mechanical properties and microstructure of concrete containing multiwalled carbon nanotubes. Mater. Perform. Charact..

[77] Sakthirswaran N., Jeyamurugan A., Sophia M., Suresh P. (2020). Effect of elevated temperatures on the properties of nano alumina modified concrete containing zircon sand as fine aggregate. Rom. J. Mater..

[78] Mohammed A., Sanjayan J.G., Nazari A., Al-Saadi N.T.K. (2017). Effects of graphene oxide in enhancing the performance of concrete exposed to high-temperature. Aust. J. Civ. Eng..

[79] Reddy L.S.I., Vijayalakshmi M.M., Praveenkumar T.R. (2022). Thermal conductivity and strength properties of nanosilica and GGBS incorporated concrete specimens. Silicon.

[80] Sherif M.A. (2017). Effect of elevated temperature on mechanical properties of nano materials concrete. Int. J. Innov. Res. Sci. Eng. Technol..

[81] Shalby O.B., Elkady H.M., Nasr E.A.R., Kohail M. (2019). Assessment of mechanical and fire resistance for hybrid nano-clay and steel fibres concrete at different curing ages. J. Struct. Fire Eng..

[82] Dahish H.A., Almutairi A.D. (2023). Effect of elevated temperatures on the compressive strength of nano-silica and nano-clay modified concretes using response surface methodology. Case Stud. Constr. Mater..

[83] Nijland T., Larbi J. (2010). Microscopic Examination of Deteriorated Concrete in Non-Destructive Evaluation of Reinforced Concrete Structures: Deterioration Processes and Standard Test Methods.

[84] Bensted J., Rbrough A., Page M. (2007). Chemical Degradation of Concrete in Durability of Concrete and Cement Composites.

[85] Dyer T. (2017). Influence of cement type on resistance to organic acids. Mag. Concr. Res..

[86] Dong Z., Wan Y., Wang P., Chen Z., He X., Hui X. (2022). Effect of long-term acid attack on impermeability and microstructure of compacted cement-bound soils. Environ. Technol..

[87] Zivica V., Bajza A. (2001). Acidic attack of cement based materials—A review. Constr. Build. Mater..

[88] Mariam Ninan C., Ajay A., Ramaswamy K.P., Thomas A.V., Bertron A. (2020). A critical review on the effect of organic acids on cement-based materials. IOP Conf. Ser. Earth Environ. Sci..

[89] Diab A.M., Elyamany H.E., Abd Elmoaty A.E.M., Sreh M.M. (2019). Effect of nanomaterials additives on performance of concrete resistance against magnesium sulfate and acids. Constr. Build. Mater..

[90] Mahdikhani M., Bamshad, Shirvani F. (2018). Mechanical properties and durability of concrete specimens containing nano silica in sulfuric acid rain condition. Constr. Build. Mater..

[91] Sujay H., Nair N.A., Sudarsana Rao H., Sairam V. (2020). Experimental study on durability characteristics of composite fiber reinforced high-performance concrete incorporating nanosilica and ultra fine fly ash. Constr. Build. Mater..

[92] Praveenkumar T., Vijayalakshmi M., Meddah M. (2019). Strengths and durability performances of blended cement concrete with TiO_2_ nanoparticles and rice husk ash. Constr. Build. Mater..

[93] Cao R., Yang J., Li G., Zhou Q., Niu M. (2023). Durability performance of multi-walled carbon nanotube reinforced ordinary Portland/calcium sulfoaluminate cement composites to sulfuric acid attack at early stage. Mater. Today Commun..

[94] Piasta W. (2017). Analysis of carbonate and sulphate attack on concrete structures. Eng. Fail. Anal..

[95] Elahi M.M.A., Shearer C.R., Naser Rashid Reza A., Saha A.K., Khan M.N.N., Hossain M.M., Sarker P.K. (2021). Improving the sulfate attack resistance of concrete by using supplementary cementitious materials (SCMs): A review. Constr. Build. Mater..

[96] Singh G., Saini B. (2022). Nanomaterial in cement industry: A brief review. Innov. Infrastruct. Solut..

[97] Wang Y., Zeng D., Ueda T., Fan Y., Li C., Li J. (2021). Beneficial effect of nanomaterials on the interfacial transition zone (ITZ) of non-dispersible underwater concrete. Constr. Build. Mater..

[98] Vijayabhaskar A., Shanmugasundaram M. (2018). Enhancing the durability properties of concrete prepared with multiwalled carbon nanotubes. Electron. J. Struct. Eng..

[99] Reshma T.V., Manjunatha M., Bharath A., Tangadagi R.B., Vengala J., Manjunatha L. (2021). Influence of ZnO and TiO_2_ on mechanical and durability properties of concrete prepared with and without polypropylene fibers. Materialia.

[100] Moslemi A.M., Khosravi A., Izadinia M., Heydari M. (2013). Application of nano silica in concrete for enhanced resistance against sulfate attack. Adv. Mater. Res..

[101] Sathe S., Zain Kangda M., Amaranatha G. (2022). Resistance against sulphate attack in concrete by addition of nano alumina. Mater. Today Proc..

[102] Vishwakarma V., Uthaman S., Dasnamoorthy R., Kanagasabai V. (2020). Investigation on surface sulfate attack of nanoparticle-modified fly ash concrete. Environ. Sci. Pollut. Res..

[103] Wang T., Cao L., Zhang F., Luo J., Jiang S., Chu H., Jiang L. (2022). Reduction of SO_4_^2−^ and Cl^−^ migration rates and degradation of silica nanoparticles incorporated cement pastes exposed to co-existence of sulfate, chloride and electric fields. Constr. Build. Mater..

[104] Zhu F., Ma Z., Zhang M. (2020). Chloride penetration into concrete under the coupling effects of internal and external relative humidity. Adv. Civ. Eng..

[105] Hu J., Zhang S., Chen E., Li W. (2022). A review on corrosion detection and protection of existing reinforced concrete (RC) structures. Constr. Build. Mater..

[106] Li D., Li L.Y., Wang X. (2019). Chloride diffusion model for concrete in marine environment with considering binding effect. Mar. Struct..

[107] Singh L., Bhattacharyya S., Sharma U., Mishra G., Ahalawat S. Microstructure improvement of cementitious systems using nanomaterials: A key for enhancing the durability of concrete. Proceedings of the Ninth International Conference on Creep, Shrinkage, and Durability Mechanics (CONCREEP-9).

[108] Rezakhani D., Jafari A., Hajabassi M. (2023). Durability, mechanical properties and rebar corrosion of slag-based cement concrete modified with graphene oxide. Structures.

[109] Liu C., Hunag X., Wu Y.Y., Deng X., Zheng Z., Yang B. (2022). Studies on mechanical properties and durability of steel fiber reinforced concrete incorporating graphene oxide. Cem. Concr. Compos..

[110] Zhang M., Du L., Li Z., Xu R. (2023). Durability of marine concrete doped with nanoparticles under joint action of Cl^-^ erosion and carbonation. Case Stud. Constr. Mater..

[111] Joshaghani A., Balapour M., Mashhadian M., Ozbakkaloglu T. (2020). Effects of nano-TiO_2_, nano-Al_2_O_3_, and nano-Fe_2_O_3_ on rheology, mechanical and durability properties of self-consolidating concrete (SCC): An experimental study. Constr. Build. Mater..

[112] Wang T., Xu J., Meng B., Peng G. (2020). Experimental study on the effect of carbon nanofiber content on the durability of concrete. Constr. Build. Mater..

[113] Li C. (2021). Chloride permeability and chloride binding capacity of nano-modified concrete. J. Build. Eng..

[114] Li T., Liu X., Wei Z., Zhao Y., Yan D. (2021). Study on the wear-resistant mechanism of concrete based on wear theory. Constr. Build. Mater..

[115] Yazıcı, İnan G. (2006). An investigation on the wear resistance of high strength concretes. Wear.

[116] Adewuyi A.P., Sulaiman I.A., Akinyele J.O. (2017). Compressive strength and abrasion resistance of concretes under varying exposure conditions. Open J. Civ. Eng..

[117] Li H., Zhang M.H., Ou J.P. (2006). Abrasion resistance of concrete containing nano-particles for pavement. Wear.

[118] Wang D., Zhang W., Ruan Y., Yu X., Han B. (2018). Enhancements and mechanisms of nanoparticles on wear resistance and chloride penetration resistance of reactive powder concrete. Constr. Build. Mater..

[119] Ghoddousi P., Zareechian M., Shirzadi Javid A.A., Habibnejad Korayem A. (2020). Microstructural study and surface properties of concrete pavements containing nanoparticles. Constr. Build. Mater..

[120] Wang L., Jin M., Guo F., Wang Y., Tang S. (2021). Pore structural and fractal analysis of the influence of fly ash and silica fume on the mechanical property and abrasion resistance of concrete. Fractals.

[121] Polat R., Demirboğa R., Karagöl F. (2017). The effect of nano-MgO on the setting time, autogenous shrinkage, microstructure and mechanical properties of high performance cement paste and mortar. Constr. Build. Mater..

[122] Zhang J. (2022). Recent advance of MgO expansive agent in cement and concrete. J. Build. Eng..

[123] Wang L., Zeng X., Li Y., Yang H., Tang S. (2022). Influences of MgO and PVA fiber on the abrasion and cracking resistance, pore structure and fractal features of hydraulic concrete. Fractal Fract..

[124] Yazdchi M., Foroughi Asl A., Talatahari S., Gandomi A.H. (2021). Evaluation of the mechanical properties of normal concrete containing nano-MgO under freeze–thaw conditions by evolutionary intelligence. Appl. Sci..

[125] Ye Q., Kaikai Yu K., Zenan Zhang Z. (2015). Expansion of ordinary Portland cement paste varied with nano-MgO. Constr. Build. Mater..

[126] Moradpour R., Taheri-Nassaj E., Parhizkar T., Ghodsian M. (2014). The effects of nanoscale expansive agents on the mechanical properties of non-shrink cement-based composites: The influence of nano-MgO addition. Compos. Part B Eng..

[127] Ye Y., Liu Y., Shi T., Hu Z., Zhong L., Wang H., Chen Y. (2021). Effect of nano-magnesium oxide on the expansion performance and hydration process of cement-based materials. Materials.

